# Multimodal tumor thermal therapy enhances antitumor immunity by expanding tumor-reactive CX3CR1⁺GPR56⁺ T cells in hepatocellular carcinoma

**DOI:** 10.7150/thno.127962

**Published:** 2026-02-26

**Authors:** Shicheng Wang, Ying Wang, Yan Zhang, Zelu Zhang, Haozhe Huang, Lichao Xu, Yuankai Hao, Yue Lou, Ke Wang, Wentao Li, Ping Liu, Lisa X. Xu, Bing Su

**Affiliations:** 1 Med-X Research Institute, School of Biomedical Engineering, Shanghai Jiao Tong University, Shanghai, China.; 2 Department of Interventional Radiology, Fudan University Shanghai Cancer Center, Shanghai, China.; 3 Department of Medical Oncology, Fudan University Shanghai Cancer Center, Shanghai, China.; 4 Shanghai Institute of Immunology, Department of Immunology and Microbiology, and the Ministry of Education Key Laboratory of Cell Death and Differentiation, Shanghai Jiao Tong University School of Medicine, Shanghai, China.

## Abstract

**Rationale:**

Tumor local ablation could facilitate the release of tumor antigens, thereby activating systemic antitumor immunity. Nevertheless, clinical observations have indicated that the systemic response induced by conventional local ablation methods is rather transient, weak, and insufficient to induce protective immunity. Multimodal tumor thermal therapy (MTT), a novel local ablation technology that involves liquid nitrogen freezing followed by radiofrequency heating, has been suggested to stimulate robust and sustained antitumor immunity. However, in patients with hepatocellular carcinoma (HCC), how MTT promotes the patients' antitumor immunity remains unknown.

**Methods:**

In this study, we enrolled four patients to receive MTT and three patients to receive radiofrequency ablation (RFA), aiming to explore the mechanism by which MTT promotes antitumor immunity.

**Results:**

We found that MTT significantly prolonged the patients' PFS compared with RFA and identified a novel T cell subset characterized by CX3CR1 and GPR56 that specifically correlated with the efficacy of MTT. MTT elicited a significant increase in CX3CR1^+^GPR56^+^ T cells and a concomitant decrease in regulatory T cells in PBMCs compared with the samples obtained from patients following RFA. CX3CR1^+^GPR56^+^ T cells express high levels of cytotoxic molecules and share TCR sequences with tumor-reactive-like T cells in tumors, and the degree of increase in the proportion of these cells is positively correlated with the PFS of patients. Compared to RFA, MTT significantly induced release of damage-associated molecular patterns (including extracellular DNA and heat shock protein 70), more effectively promoted dendritic cell maturation, and strengthened their interaction with CX3CR1^+^GPR56^+^ effector T cells via MHC I-TCR. Meanwhile, MTT diminished the interactions between DCs and Tregs through LGALS9-CD45 axis, leading to a reduction in peripheral Tregs.

**Conclusions:**

These findings reveal the mechanism by which MTT promotes antitumor immunity in patients with HCC and warrant further investigation in large-scale clinical studies.

## Introduction

Primary liver cancer ranks as the sixth most common cancer and was the third leading cause of cancer-related death worldwide in 2020 1. Hepatocellular carcinoma (HCC) represents 90% of all primary liver cancers 2. Moreover, approximately 72% of HCC cases occur in Asia, with China having the largest number of liver cancer patients in the world 3.

For patients with intermediate-stage HCC, local therapy has been identified as the preferred treatment option 2. Ablation therapy is the main nonsurgical local treatment. Currently, the most common ablation techniques employed in clinical practice include cryoablation, radiofrequency ablation (RFA) and microwave ablation (MWA) 4. Ablative therapy induces local and systemic immune responses that may eliminate distant metastatic lesions, a phenomenon known as the “abscopal effect” 5. The DAMPs released post-ablation orchestrate a cascade response, facilitating conventional type I dendritic cells (cDC1s) to phagocytose tumor debris, process released antigens, and present them on MHC-I molecules, which subsequently activates naive CD8⁺ T cells in the draining lymph nodes 6-8. However, it has been suggested that ablation-induced immune responses are typically weak and insufficient to induce consistent protective antitumor immunity 5. The type and quantity of tumor antigens and DAMPs released are influenced by the ablation modality and conditions, which ultimately lead to the induction of different immune responses 4. Therefore, it is theoretically possible to maximize the induced antitumor immune response by optimizing the ablation modality and ablation conditions.

Multimodal tumor thermal therapy (MTT) is an innovative tumor ablation technique that was developed by our research team and approved by the China National Medicine Products Administration (No. 20233010773). It combines RFA, cryoablation, and uses mild temperatures to ablate tumors 9, 10. Preclinical animal studies suggested that MTT resulted in the release of more DAMPs and tumor antigens compared to RFA, thereby remodeling the tumor immune environment 11, 12 and inducing Th1-dominated CD4^+^ T cells antitumor immunity 13-15. Previous clinical studies have shown that MTT prolongs progression-free survival in patients with colorectal liver metastases (CRCLM) and triggers robust T cell-mediated antitumor responses compared with RFA 9, 10. Notably, effective antitumor immunity critically depends on the activation and expansion of tumor-reactive T cells 16. Among these, circulating CX3CR1⁺ T cells have emerged as a key subset associated with improved clinical outcomes in various immunotherapies 17-19. Nevertheless, the precise mechanism by which MTT induces an antitumor immune response in patients, particularly its impact on specific functional T cell subsets such as tumor-reactive T cells, remains unclear.

To investigate the underlying mechanisms, this study included four HCC patients treated with MTT and three HCC patients who received RFA. Single-cell RNA sequencing (scRNA-seq) and flow cytometry were performed on pre-treatment tumor biopsy specimens and serially collected peripheral blood mononuclear cell (PBMC) samples. Moreover, subcutaneous Hepa1-6 HCC mouse models were used to validate the clinical relevance of these findings. We found that MTT but not RFA, decreased Tregs levels but increased the proportion of CX3CR1^+^GPR56^+^ T cells in PBMCs, a cell population characterized by elevated expression of cytotoxic molecules and containing a substantial number of Ttr-like cells. The extent of this cellular expansion showed a significant positive correlation with the patients' PFS. Overall, this study demonstrates that MTT can exert in situ vaccine effects, simultaneously reducing tumor burden and activating anti-tumor immunity in HCC patients.

## Results

### MTT prolongs PFS in HCC patients

**Figure 1:**
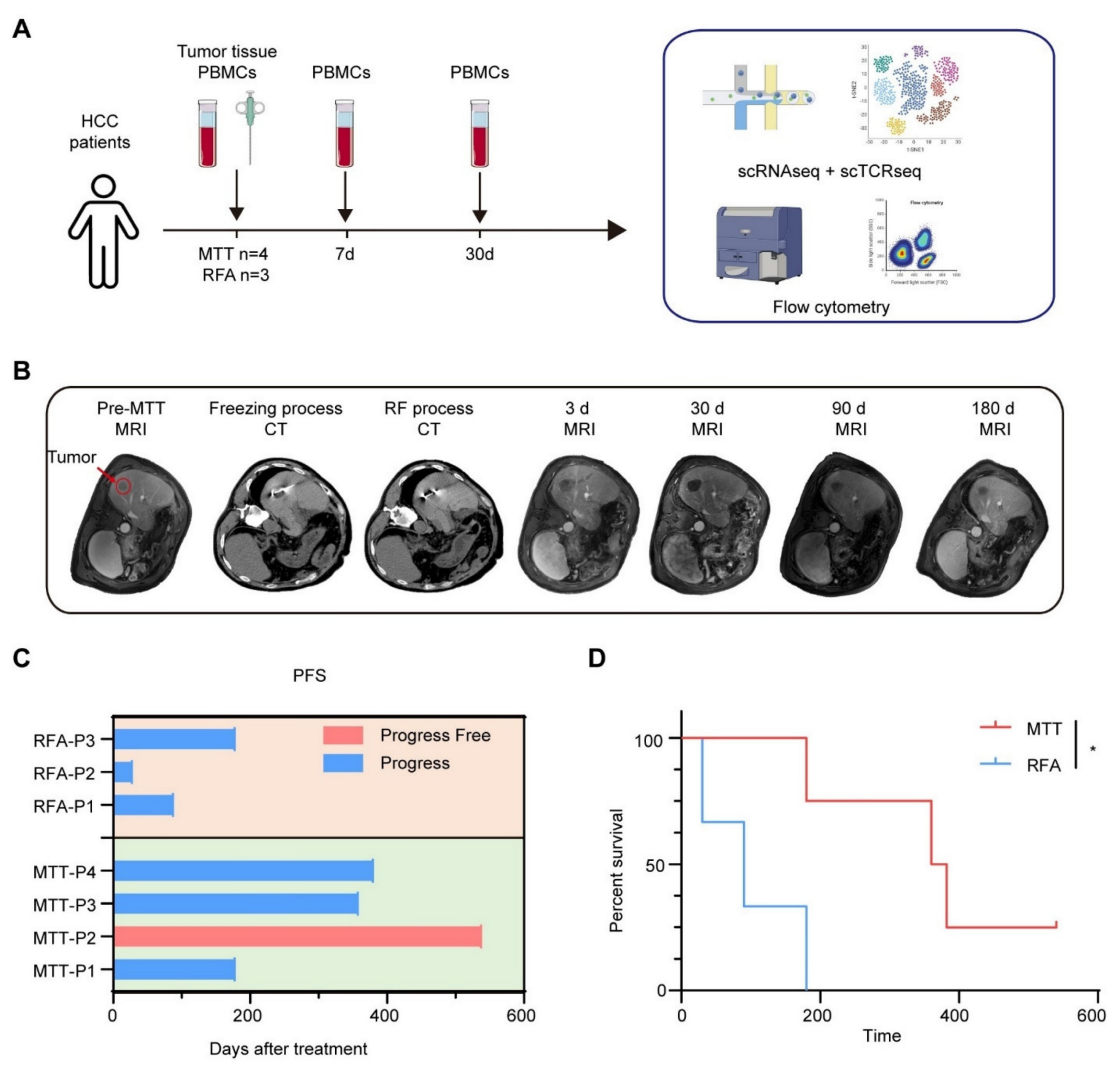
** Experimental protocol and patient survival outcomes.** (A) Experimental schema showing the time of samples collection and the study design. (B) Representative graphs of CT images and MRI at pre-treatment, on-treatment, and post-treatment series times. (C) The progression-free survival time for each patient (D) Kaplan-Meier curves for PFS. *P < 0.05.

A prospective clinical study was conducted to investigate the stimulation of anti-tumor immunity in HCC patients after MTT. A total of seven HCC patients were enrolled in the study. All patients had undergone hepatectomy for liver tumors. Some patients also received transcatheter arterial chemoembolization (TACE), targeted therapy (tyrosine kinase inhibitors) or immunotherapy (PD-1 inhibitors) before recruitment (Table S1). Four patients received MTT and three patients received RFA as controls. Preoperative tumor tissues and PBMCs, and PBMCs at 7 days and 30 days after MTT or RFA, were collected from each patient and subjected to scRNA-seq and cryopreservation for subsequent flow cytometry analysis (Figure 1A). All enrolled patients underwent triphasic contrast-enhanced MRI evaluation at scheduled time points following treatment (Figure 1B). Three of four patients who received MTT had a PFS of more than one year. In contrast, all three patients who received RFA exhibited disease progression within 180 days (PFSs of 30 d, 90 d, and 180 d for the three patients, respectively) (Figure 1C). This finding suggested that MTT prolonged PFS in HCC patients compared with RFA, as illustrated in Figure 1D.

### MTT remodeled the immune environment of HCC patients

Single-cell RNA sequencing was used to characterize the immune landscape and dynamics of different types of immune cells in patients before and after MTT. After quality filtering, single-cell transcriptome data for 247,022 high-quality immune cells were obtained. Through dimensionality reduction and clustering, subsequent analysis identified several clusters including classical monocytes (C2_Mono, C4_Mono, C9_Mono, C17_Mono), nonclassical monocytes (C7_NCMono), classical dendritic cells (C14_cDC), plasmacytoid dendritic cells (C16_pDC), CD4^+^ T cells (C0_CD4), CD8^+^ T cells (C3_CD8, C5_CD8), NK and NKT cells (C1_NK, C18_NK), B cells (C8_Bcell), plasma cells (C15_plasma), stromal cell types (C11_EC), hepatocellular carcinoma cells (C10_HCC), platelets (C13_Platelet) and a group of proliferating cells (C12_MKI67), on the basis of marker gene expression (Figure S1A-B). No statistically significant difference was observed in the immune cell profiles of TME and PBMCs between patients in the MTT group and those in the RFA group prior to treatment. (Figure S1C).

We subsequently performed functional characterization of these cellular populations. First, the expression of DC maturation-associated markers, including cytokines *IL12A*, *IL18*, *IL23A*, co-stimulatory molecules *CD86*, *TNFSF9*, T-cell chemokines *CXCL9*, *CXCL11*, and interferon-stimulated genes, were significantly increased after MTT but not after RFA (Figure S2A), indicating that DCs underwent significant maturation following MTT. In addition, we assessed the activation of lymphocytes after MTT. Previously, we reported that MTT rapidly promoted NK cell activation in a B16F10 model 20. In this study, the expression of *GZMB* and *PRF1* in NK and NKT cells from PBMCs was also significantly upregulated in HCC patients after MTT compared with baseline (Figure S2B).

However, following RFA, there was no elevation in the expression of GZMB and PRF1, and in fact, PRF1 expression decreased significantly (Figure S2B). Subsequently, an analysis of B cells was conducted in peripheral blood. Gene set enrichment analysis (GSEA) based on gene ontology biological process (GOBP) terms revealed that the pathways associated with humoral immune response, B cell mediated immunity, phagocytosis, B cell activation and B cell receptor signaling the were significantly enriched in B cells from patients who received MTT compared with those who received RFA (Figure S2C). Next, subclustering analysis of a total of 19,137 B cells revealed three clusters of naïve B cells (TCL1A_1, TCL1A_2 and TCL1A_3) that highly expressed *TLC1A*, *IL4R*, and *FCER2*; two cluster of memory B cells (B_AIM2_1, B_AIM2_2) signature of *AIM2*, *SCIMP* and *TNFRSF13B*; and two clusters of plasma cells (Plasma cell and Plasmablast) that highly expressed the genes *MZB1*, *XBP1*, and *JCHAIN* and genes encoding immunoglobulins (Figure S2D). Compared with that at baseline, the proportion of naïve B cells was decreased, while the proportion of plasma cells was dramatically increased 7 days after MTT (Figure S2E). However, although the proportion of plasma cells was increased in the patients in the RFA group, it was much lower than that observed in the patients in the MTT group (Figure S2E). These results suggest that MTT induced a stronger host humoral immune response compared to RFA. Furthermore, GSEA based on the GOBP gene set demonstrated that pathways related to cellular toxicity and effector function were most significantly upregulated by T cells in the PBMCs of patients following MTT (Figure S2F). However, these pathways were downregulated after RFA (Figure S2F). In summary, MTT, but not RFA, effectively remodels the systemic immune environment and thus enhances anti-tumor immune responses.

### MTT increases the proportion of peripheral CX3CR1^+^GPR56^+^T cells

**Figure 2:**
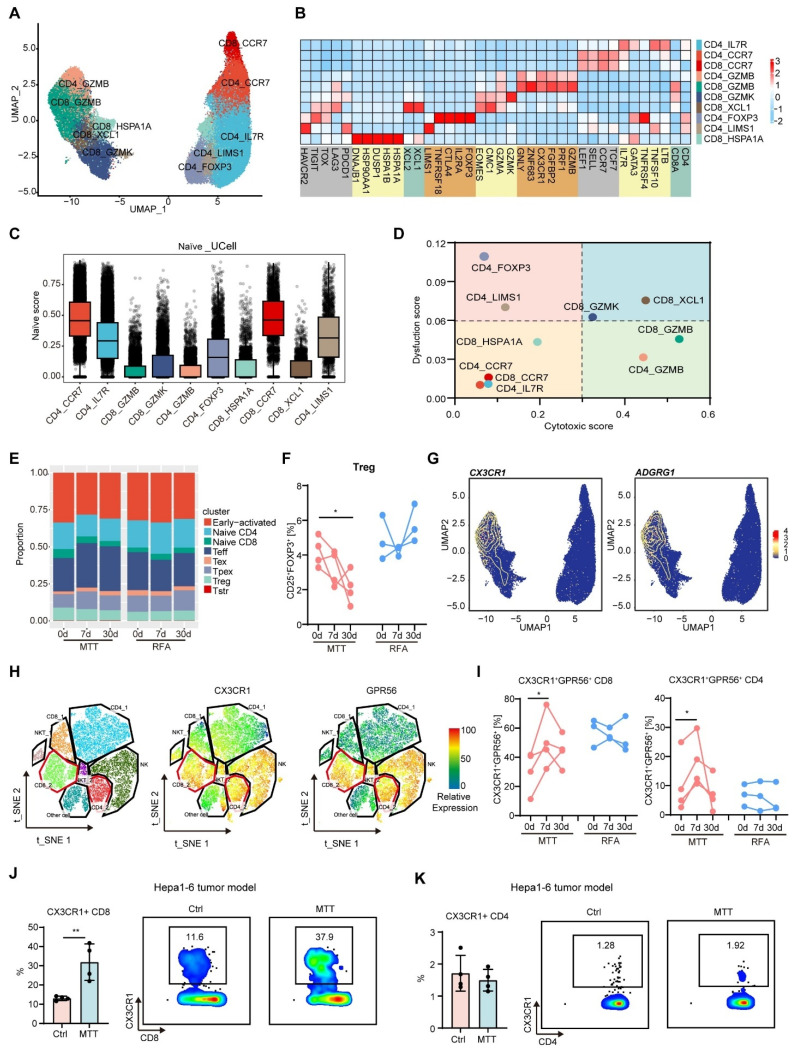
**The proportion of T_eff_ in the PBMCs of patients was increased after receiving MTT rather than RFA.** (A) UMAP visualization of T cells transcriptomes in tumor and PBMCs from HCC patients. (B) Heatmap illustrated the expression levels of the signature gene for each cluster in T cells. (C-D) The naïve, dysfunctional and cytotoxic score were calculated by R package UCell. (E) The proportion of each cluster in PBMCs was determined before and at 7 and 30 days after MTT and RFA. (F) The proportion of Tregs (CD25^+^FOXP3^+^) was measured by flow cytometry. n = 4 for MTT and n = 3 for RFA. Paired student's t-test was used. *P < 0.05. (G) The expression levels of CX3CR1 and ADGRG1 in T cells. The yellow lines represent the spatial density of the cells expressing the given gene higher than the mean level of expression. (H) t-SNE visualization of T cells and NK cells subsets and the expression of CX3CR1 and CPR56 measured by using flow cytometry. (I) The proportion of CX3CR1^+^GPR56^+^ cells in CD8^+^ T cells and CD4^+^ T cells from patients' PBMCs. n = 4 for MTT and n = 3 for RFA. Paired student's t-test was used. *P < 0.05. (J-K) The proportion of CX3CR1^+^ cells in CD8^+^ T cells and CD4^+^ T cells from blood in Hepa1-6 HCC mouse model before and 7 days after MTT. n = 4. Unpaired student's t-test was used. Error bars represent the standard deviation. **P < 0.01.

In our previous preclinical study, we reported that MTT induced Th1 subpopulation-dominated anti-tumor immunity and enhanced the memory CD8^+^ T-cell response 21. Therefore, we next sought to investigate whether MTT could induce similar anti-tumor immunity in patients. After the removal of unconventional T cells, 59,642 T cells were subclustered into 10 subsets (Figure 2A and Table S2). First, distinct functional and phenotypic states of CD4^+^ T cells were distinguished according to their gene expression signatures: CD4_CCR7 (*CCR7, TCF7, SELL* and *LEF1*) for naïve CD4^+^ T cells; CD4_IL7R (*IL7R, GPR138, ANXA1* and *CD69*) for early-activated CD4^+^ T cells 22; CD4_LIMS1 (*LIMS1, PDCD1* and *HAVCR2*), CD4_FOXP3 (*FOXP3, IL2RA, CTLA4* and *TNFRSF18*) for regulatory T cells (Tregs) 23, and CD4_GZMB (*GZMB, PRF1* and *GNLY*) for effector or cytotoxic CD4^+^ T cells (Figure 2, B). Naïve CD4^+^ T cells (CD4_CCR7) and early-activated CD4^+^ T cells had the highest naïve score and low dysfunction and cytotoxic scores (Figure 2C-D) Moreover, five states of CD8^+^ T cells were identified and annotated on the basis of their featured markers: (1) CD8_CCR7, (2) CD8_XCL1, (3) CD8_GZMK, (4) CD8_HSPA1A and (5) CD8_GZMB (Figure 2B). Like CD4_CCR7 cells, CD8_CCR7 cells, which also express the naïve T cell markers *CCR7, TCF7, SELL* and *LEF1*, had high naïve scores (Figure 2B-C). CD8_HSPA1A cells expressed stress-related heat shock genes (*HSPA1A, HSPA1B* and *HSP90AA1*) and stress response genes (*DUSP1* and *DNAJB1*), similar to the previously described cluster with a unique stress response state (T_str_) 24 (Figure 2B). T_str_ cells, which are near or within hypoxic conditions and play a potential role in immunotherapy resistance 24, had a low dysfunction score and a low cytotoxicity score, indicating the loss of effector function (Figure 2D). CD8_GZMK and CD8_XCL1 cells were characterized by elevated expression of *GZMK, CMC1*, coinhibitory molecules or exhaustion promoting molecules (*PDCD1, TIGIT* and *TOX*) and HLA-II genes and had high dysfunction scores, similar to previously reported exhausted T-cell subpopulations (T_ex_) 25. High expression of *GZMK, CMC1, EOMES* and coinhibitory molecules is associated with dysfunctional T cells 26. Moreover, the elevated expression of HLA-II genes is associated with T cell activation and reduced cytolytic activity 27. CD8_GZMK cells expressed low levels of cytolytic genes, such as GZMB and PRF1, and had a relatively low cytotoxic score (Figure 2B-D). The CD8_GZMB cluster presented increased expression of *GZMB, PRF1, GNLY* and *KLRD1* and high cytotoxicity and low dysfunction scores, indicating that these cells are highly cytotoxic (Figure 2B-D). Both CD4_GZMB and CD8_GZMB cells presented increased expression of *ZNF683*, a marker of tissue-resident memory T (Trm) cells 28, and directly regulated the key pathways involved in T cell cytotoxicity and activation 29. According to the global transcriptional profile, CD4_GZMB and CD8_GZMB cells exhibited stronger effector functions than cells in the other clusters, similar to previously reported effector T cells (T_eff_) 25.

We subsequently examined the changes in T cell subsets in the PBMCs of patients before and after treatment. Compared with those before MTT, the PBMCs of patients who received MTT exhibited a reduced proportion of Tregs (CD4_FOXP3 and CD4_LIMS1) (Figure 2E). However, the proportions of Tregs in the PBMCs of patients who received RFA remained unchanged (Figure 2E). By using flow cytometry, we also observed a gradual decrease in the proportion of FOXP3^+^CD25^+^ Tregs within the CD4^+^ T cell population in patients on day 7 and 30 following MTT, compared with the baseline state (Figure 2F). However, the percentage of Tregs remained unchanged in patients who received RFA (Figure 2F).

Furthermore, our findings showed that MTT led to a significant increase in the proportions of T_eff_ (CD8_GZMB and CD4_GZMB) compared with RFA (Figure 2E), suggesting that MTT induced a stronger T cell response. Accordingly, we attempted to identify these cells via flow cytometry. Through differentially expressed gene analysis, we found that the CD8_GZMB and CD4_GZMB clusters expressed *CX3CR1* and *ADGRG1* (gene that encode GPR56), which were rarely expressed in other subpopulations (Figure 2G). Subsequently, CX3CR1^+^ and GPR56^+^ T cells were detected in T cells from patient PBMCs (clusters CD8_2 and CD4_2). Flow cytometry analysis revealed that these two molecules were highly co-expressed on T cells (Figure 2H). More importantly, the proportion of CX3CR1^+^GPR56^+^ cells among CD8^+^ and CD4^+^ T cells in the PBMCs of patients after MTT but not RFA was significantly greater than that at baseline (Figure 2I). The results indicated that MTT reduced the proportion of immunosuppressive Tregs but increased the proportion of T-cell subsets with effector functions in PBMCs.

Next, we established a subcutaneous Hepa1-6 mouse hepatocellular carcinoma tumor model, and performed MTT. Owing to the coexpression property of CX3CR1 with GPR56, we only assayed CX3CR1^+^ T cells. Similarly, the proportion of CX3CR1^+^ CD8^+^ T cells in the blood of the subcutaneous Hepa1-6 mouse hepatocellular carcinoma tumor model that received MTT was significantly greater than that in the blood of the untreated mice (Figure 2J). However, the proportion of CX3CR1^+^CD4^+^ T cells remained unchanged (Figure 2K), possibly due to their lower levels in the blood of mice. Collectively, these results suggest that MTT can significantly increase the number of CX3CR1^+^ T_eff_ cells in the peripheral blood.

We observed that CX3CR1^+^GPR56^+^ T_eff_ was increased at 7 days post-MTT, but decreased by day 30 (Figure 2I), then we hypothesized that these T_eff_ might undergo migration into the tumor. GSEA based on KEGG gene sets revealed that both CD8_GZMB and CD4_GZMB T_eff_ were significantly enriched in pathways related to cell motility and migration (Figure S3A). Accordingly, a Hepa1-6 mouse model was established, and a transwell system was used to investigate the migratory capacity of CX3CR1^-^ and CX3CR1^+^ CD8^+^ T cells from mice following MTT (Figure S3B). In the absence of tumor cells in the lower chamber, CX3CR1⁺ T cells exhibited significantly greater migration into the lower compartment compared to CX3CR1⁻ T cells, suggesting their enhanced intrinsic motility (Figure S3C). The addition of tumor cells markedly enhanced the migratory capacity of T cells into the lower compartment, with CX3CR1^+^ CD8^+^ T cells exhibiting significantly elevated migratory activity compared to CX3CR1^-^ CD8^+^ T cells (Figure S3C). To further validate in vivo whether CX3CR1^+^GPR56^+^ T_eff_ migrate toward tumors, we established a Hepa1-6 bilateral tumor model. At 7 days following MTT treated the right-side tumor, we quantified the number of CX3CR1^+^ T cells in the left-side tumor. We found that, compared to the untreated group, MTT significantly promoted the intratumoral infiltration of both CX3CR1^+^ CD4^+^ T cells and CX3CR1^+^ CD8^+^ T cells in the left-side tumor (Figure S3D). These results demonstrate that MTT-induced CX3CR1^+^GPR56^+^ T_eff_ possess the capacity to migrate into tumors.

### Elevated levels of CX3CR1^+^GPR56^+^ effector T cells are associated with improved patient prognosis

After MTT, there was a significant increase in the proportion of CX3CR1^+^GPR56^+^ T cells in the PBMC population, with a low dysfunction score and high expression of cytotoxicity-associated molecules, which prompted us to further investigate these cells.

**Figure 3:**
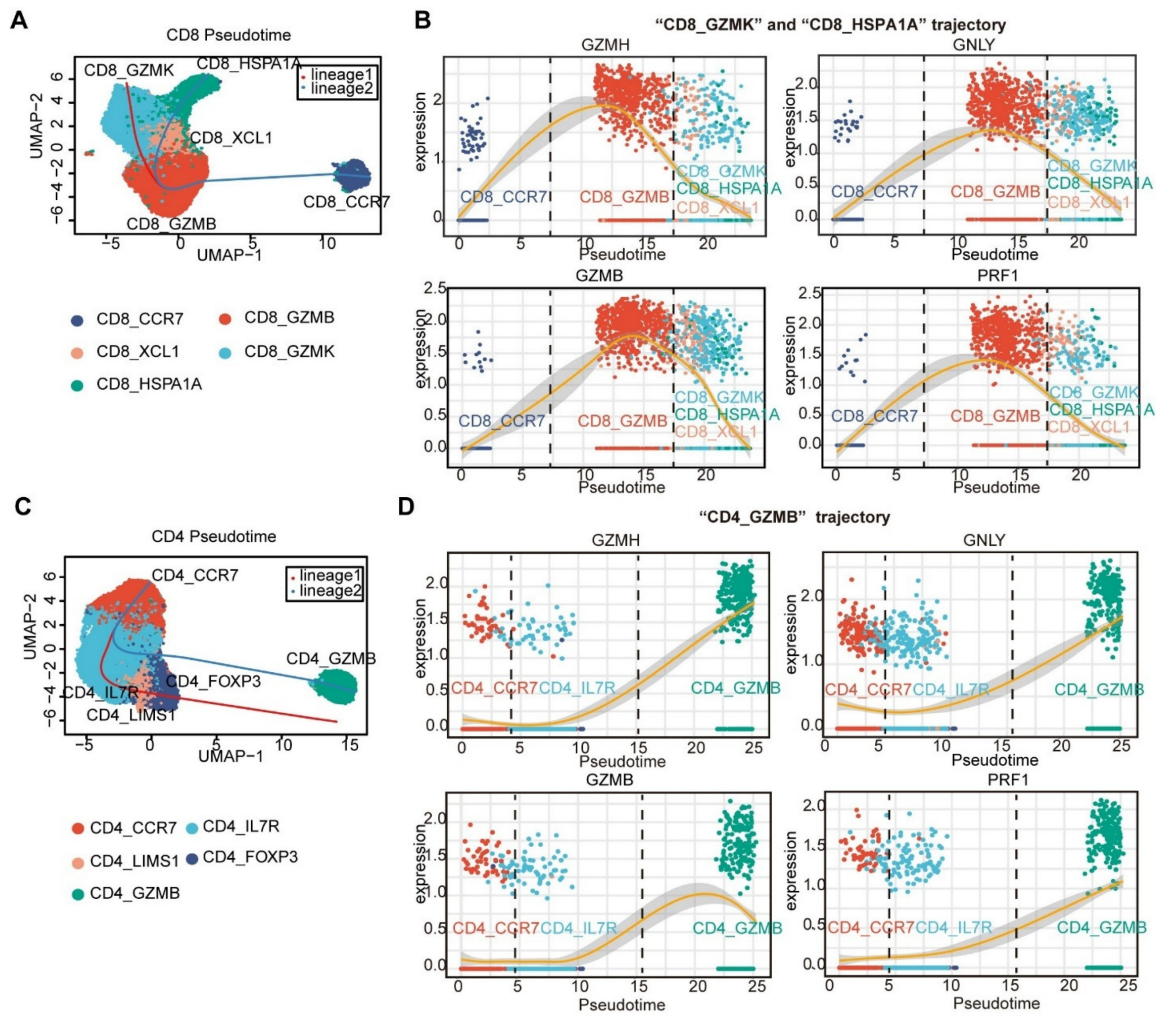
** Pseudotime trajectories of CD4⁺ and CD8⁺ T cells.** The Slingshot R package 30 was used to reveal the pseudotime trajectories of CD8⁺ and CD4⁺ T cells (A and C), and to profile the expression of cytotoxicity-related genes along these trajectories (B and D).

Pseudotime trajectories of CD8^+^ and CD4^+^ T cells were generated via Slingshot 30. Naïve CD8^+^ T cells (CD8_CCR7) and naïve CD4^+^ T cells (CD4_CCR7) were considered the roots of the trajectory. For CD8⁺ T cells, the naïve CD8_CCR7 cluster differentiated into an effector cluster (T_eff_, CD8_GZMB). This T_eff_ cluster then diverged into two distinct lineages: one giving rise to exhausted T cells (T_ex_, CD8_GZMK) and the other to precursor exhausted T cells (T_pex_, CD8_XCL1). The T_pex_ cluster further differentiated into a stressed T cell cluster (T_str_, CD8_HSPA1A) (Figure 3A). Upon differentiation of CD8_CCR7 cluster into the CD8_GZMB cluster, a high-level expression of cytotoxic genes, including *GNLY*, *GZMH*, *GZMB*, and *PRF1*, was observed (Figure 3B). However, as the CD8 _GZMB cluster differentiates towards the T_ex_, T_pex_, and T_str_ clusters, the expression of these cytotoxicity-related genes gradually decreases ((Figure 3B). This finding is consistent with the results of previous investigations, which suggested that CX3R1^+^CD8^+^ T cells may function as a source of T_ex_ 31-33. For CD4^+^ T cells, naïve CD4^+^ T cells (CD4_CCR7) were differentiated into early-activated CD4^+^ T cells (CD4_IL7R) and then into Tregs (CD4_LIMS1 and CD4_FOXP3) and CD4_GZMB via two differentiation trajectories (Figure 3C). We found that the expression of cytotoxic genes increased along “CD4_GZMB trajectory” (Figure 3D).

**Figure 4:**
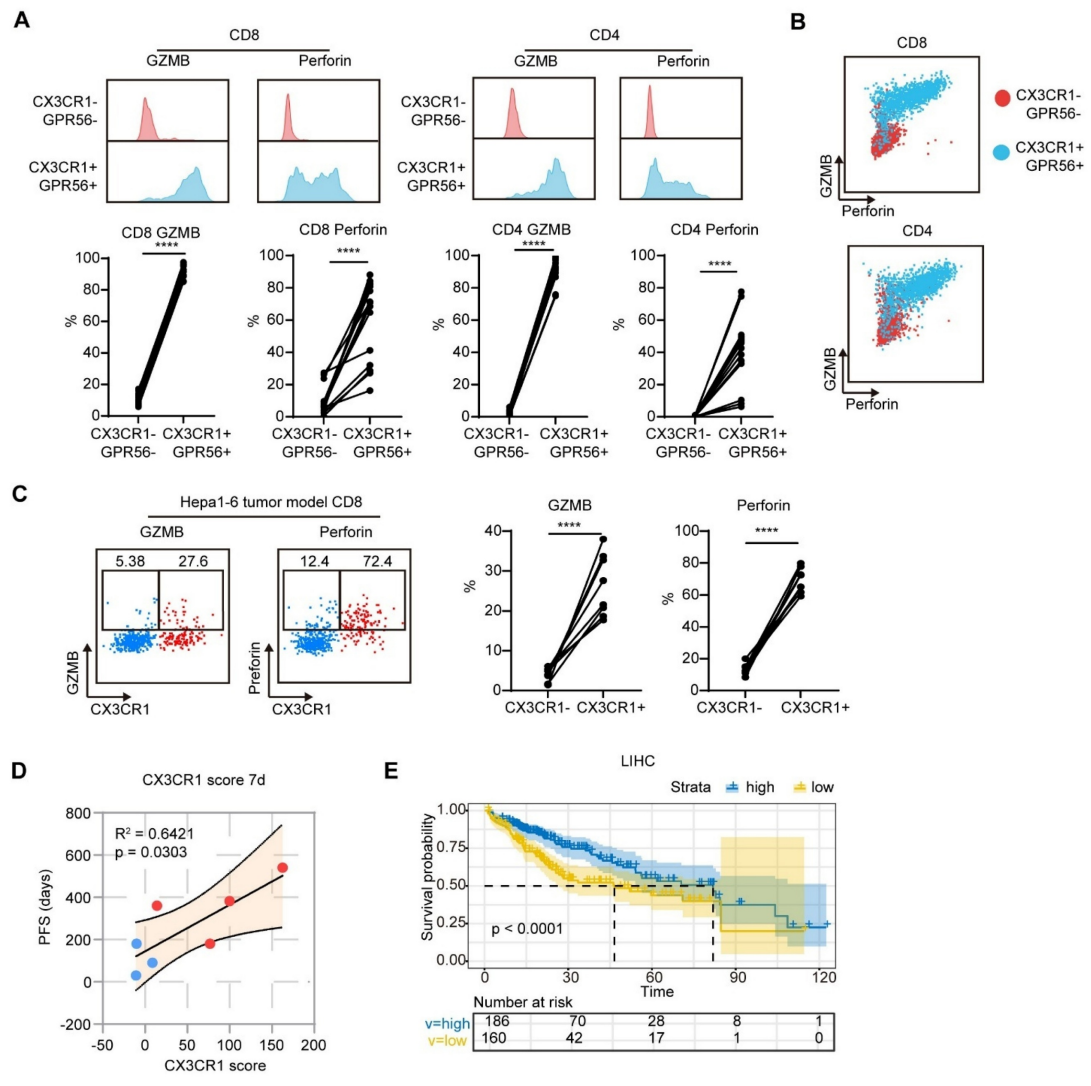
**CX3CR1^+^ T cells exhibit elevated expression of cytotoxic molecules.** (A-B) The expression of GZMB and perforin in CX3CR1^-^GPR56^-^ and CX3CR1^+^GPR56^+^ T cells in PBMCs from HCC patients was measured by flow cytometry. n = 12. Paired student's t-test was used. ****P < 0.0001. (C) The expression of GZMB and perforin in CX3CR1^-^ and CX3CR1^+^ T cells in blood from Hepa1-6 mouse models was measured by flow cytometry. n = 8. Paired student's t-test was used. ****P < 0.0001. (D) Correlation of CX3CR1 score with PFS in patients. (E) Patients in the TCGA cohort were divided into high and low CX3CR1 score groups according to the expression patterns of CD8_GZMB and CD4_GZMB subgroup gene signatures. Kaplan-Meier survival analysis was performed to compare the overall survival (OS) between the two groups.

Given that the CD8_GZMB and CD4_GZMB clusters presented pronounced expression of cytotoxicity-related genes, we explored the capacity of the CD8_GZMB and CD4_GZMB subsets to express the cytotoxic molecules by using flow cytometry. We found that CX3CR1^+^GPR56^+^ T cells expressed higher levels of granzyme B and perforin than T CX3CR1^-^GPR56^-^ cells (Figure 4A). Almost all GZMB^+^Perforin^+^ cells (over 90%) were CX3CR1^+^GPR56^+^ (Figure 4B). Similarly, CX3CR1^+^CD8^+^ T cells presented higher expression levels of granzyme B and perforin than CX3CR1^-^CD8^+^ T cells in the Hepa1-6 tumor model (Figure 4C). These findings indicate that CX3CR1^+^GPR56^+^ T cells exhibit heightened cytotoxicity.

We subsequently assessed the correlation between the mean value of the percentage change from baseline in CX3CR1^+^ cells among CD8^+^ and CD4^+^ T cells (CX3CR1 score) and patient PFS. The CX3CR1 score at 7 days after treatment was positively correlated with patient PFS (Figure 4D). In addition, patients with high expression of the gene signatures of CD8_GZMB and CD4_GZMB in the TCGA database presented a longer overall survival compared to patients with low expression of the same gene signatures (median survival: 80 months versus 46 months) (Figure 4E). Collectively, these findings indicate that the MTT-induced increase in the proportion of CX3CR1^+^GPR56^+^ T_eff_ serves as a prognostic biomarker associated with favorable clinical outcomes in HCC patients.

### CX3CR1^+^GPR56^+^ CD8^+^ T cells in blood are enriched in tumor-reactive-like T cells

To determine whether MTT-induced CX3CR1^+^GPR56^+^ CD8^+^ T cells are reactive to tumors, we furthermore compared TCR clonality of CD8^+^ T cells between tumors and blood by combining scRNA-seq and single-cell T-cell receptor sequencing (scTCR-seq). 5276 unique TCRs in the tumor microenvironment (TME) and 28767 unique TCRs in PBMCs were identified. After excluding TCR clones targeting common viruses, bacteria and autoantigens 34, TCR clones of CD8^+^ T cells with >5 clones in the TME were defined as tumor-reactive-like T (Ttr-like) cells. All the Ttr-like cell clones were unique to each patient (Figure S4A).

**Figure 5:**
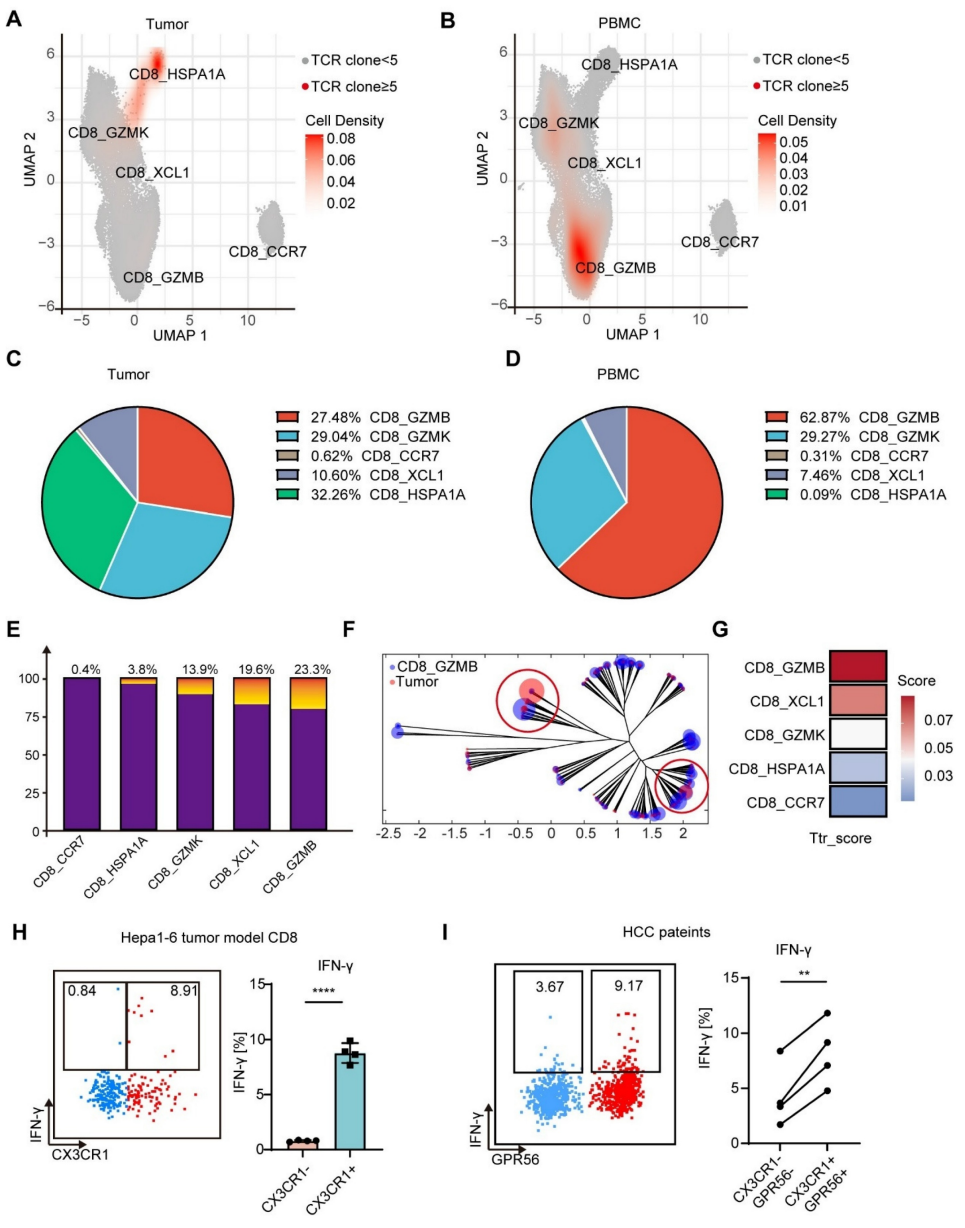
**CD8_GZMB effector T cells in blood were enriched for Ttr-like cells.** (A-B) UMAP visualization of CD8^+^ T cells transcriptomes in tumor and PBMCs from HCC patients. Ttr-like cells (TCR clonotypes with numbers > 5 in the TME) were highlighted (red dot). (C-D) Pie chart showing the distribution of subpopulations of Ttr-like cells in TME (C) and PBMCs (D). (E) Proportion of Ttr-like cells in each subpopulation of CD8^+^ T cells. The Ttr clones are colored while other clones are in purple. (F) Overlapped weighted repertoire dendrograms of repertoire in CD8_GZMB vs Tumor. (Red = Tumor repertoire. Blue = CD8_GZMB repertoire in PBMCs). (G) Ucell score of tumor-reactive CD8^+^ T cell signature across CD8^+^ T cell subsets. Ucell scores for a validated nine-gene signature (*CXCL13, CTLA4, ENTPD1, LAYN, TIGIT, BATF, HAVCR2, TNFRSF9, GZMB*) are shown as a heatmap. (H) Splenocytes from mice after MTT were co-cultured with Hepa1-6 cells for 12 h and treated with brefeldin A for 6 h. Tumor reactive T cells were measured by the intracellular IFN-γ expression. The proportion of tumor reactive T cells in CX3CR1- and CX3CR1^+^ T cells. n = 4. Paired student's t-test was used. Error bars represent the standard deviation. ****P < 0.0001. (I) Tumor single-cell suspensions depleted of leukocytes were co-cultured with autologous PBMCs from the same patient for 12 h, followed by a 6 h incubation with brefeldin A. Intracellular IFN-γ expression in CD8⁺ T cells was then analyzed by flow cytometry. n = 4. Paired student's t-test was used. **P < 0.01.

The clusters of these Ttr-like cells were subsequently analyzed. In the TME, Ttr-like cells are distributed across various subsets except for naïve T cells (CD8_CCR7), with proportions as follows: T_str_ (CD8_HSPA1A) at 32.26%, T_ex_ (CD8_GZMK and CD8_XCL) at 39.64%, and T_eff_ (CD8_GZMB) at 27.48% (Figure 5A-C). However, Ttr-like cells among PBMCs exhibited dramatically different subpopulation distributions. Among the PBMCs, more than half of the Ttr-like cells belonged to the CD8_GZMB subset (62.87%) (Figure 5B-D). We subsequently quantified the proportions of Ttr-like cells within each T cell subpopulation of PBMCs. The results indicated that 23.3% of the CD8_GZMB T_eff_ were Ttr-like (Figure 5E). Sequences with a high degree of homology and similar sequence features may represent TCRs that recognize the same targets. We therefore analyzed the similarity of the structural TCR repertoire of different subpopulations of T cells in PBMCs and intratumor T cells using ImmunoMap 35. Compared with those of other clusters, the structural clones of the CD8_GZMB subsets in PBMCs were more shared with those amplified within the tumor, as they existed on the same or similar branches in the dendrograms (Figure 5F and Figure S4B). To further characterize the tumor-reactive potential of these populations, we evaluated their enrichment for an established tumor-reactive CD8^+^ T cell signature comprising nine genes (*CXCL13*, *CTLA4*, *ENTPD1*, *LAYN*, *TIGIT*, *BATF*, *HAVCR2*, *TNFRSF9*, and *GZMB*) that exhibit high discriminative power in distinguishing tumor-reactive from bystander T cells (AUC > 0.8) 36. Using Ucell scoring analysis, we found that the CD8_GZMB subset showed the highest enrichment for this tumor-reactive signature among all CD8^+^ T cell subsets (Figure 5G).

To functionally validate the tumor reactivity of the CD8_GZMB subset, we first employed a Hepa1-6 mouse model. Spleen cells from mice receiving MTT were cocultured with Hepa1-6 cells, and tumor-reactive T cells were characterized by the expression of IFN-γ. We found that Ttr-like cells were predominantly CX3CR1^+^CD8^+^ T cells rather than CX3CR1^-^CD8^+^ T cells (Figure 5H). We next sought to confirm these findings in patient samples. Single-cell suspensions from HCC tumor tissues (after CD45⁺ leukocyte depletion) were co-cultured overnight with autologous PBMCs. Flow cytometric analysis of intracellular IFN-γ in CD8⁺ T cells showed that the CX3CR1⁺GPR56⁺CD8⁺ T-cell subset contained a significantly higher frequency of tumor-reactive cells compared to the CX3CR1^-^GPR56^-^CD8⁺ subset. Collectively, these data demonstrate that the CX3CR1⁺GPR56⁺CD8⁺ T cells (corresponding to the CD8_GZMB subset) in PBMCs are highly enriched for tumor-reactive T cells.

### MTT promotes clonal expansion of CX3CR1^+^ GPR56^+^ Ttr-like cells

**Figure 6:**
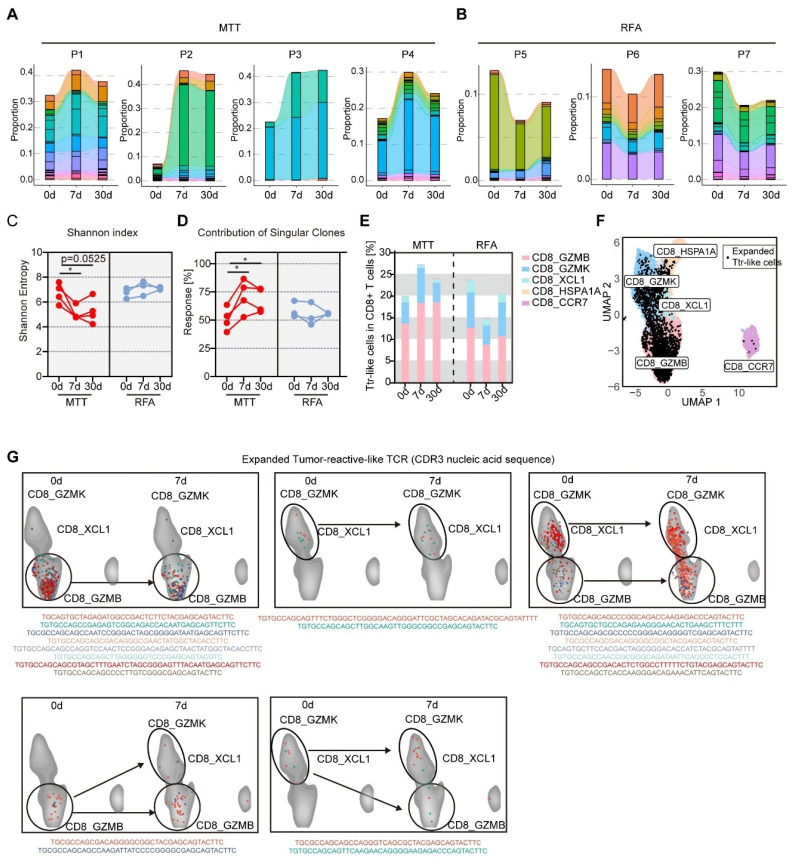
**MTT but not RFA promoted clonal expansion of CX3CR1^+^GPR56^+^Ttr-like cells.** (A-B) Clonotype tracking function was used to track the Ttr-like TCR clonotypes across different time points in PBMCs. Different colors represent different clonotypes. (C-D) Shannon index and contribution of singular clones of structural TCR repertoire were calculated by R package immunomap. n = 4 for MTT and n = 3 for RFA. Paired student's t-test was used. *P < 0.05. (E) The distribution of clonally expanded Ttr-like cell subsets was analyzed, with clonal expansion defined as TCR clonotypes showing a proportional increase of more than 1.5-fold relative to baseline. (F) UMAP visualization of clonally expanded Ttr-like cell (black dots). (G) The UMAP plot showed the shifting pattern of subset distribution of TCR clonotypes before and after MTT.

The above results suggest that CX3CR1^+^GPR56^+^CD8^+^ T_eff_ cells contain a significant proportion of Ttr-like cells. Therefore, we hypothesized that the observed increase in the proportion of T_eff_ cells after MTT likely due to the expansion of this Ttr-like cell population. Interestingly, significant amplification of Ttr-like cells at 7 days after MTT was detected in all patients who received MTT (Figure 6A). However, the percentage of Ttr-like cells in the PBMCs of patients who received RFA was lower than that at baseline (Figure 6B). Then, using ImmunoMap, we analyzed the TCR repertoire using a phylogenetic approach 35. The Shannon‒Weiner index reflects the clonal expansion of TCRs, with lower values indicating a greater degree of clonal expansion 37. We found that the Shannon‒Weiner index of the structural TCR repertoire in PBMCs was significantly reduced in patients at 7 and 30 days after MTT (Figure 6C). In contrast, in patients who underwent RFA, the Shannon‒Weiner index remained unchanged (Figure 6C). In addition, the increase in the response contributed by singular clones reflects the tendency for the immune response to be dominated by a small number of dominant clones. We found that the response contributed by singular clones was significantly increased in patients after receiving MTT but not RFA, indicating an enrichment of effective structural motifs after MTT (Figure 6D). More importantly, the proportion of Ttr-like cells within the CD8_GZMB subset, but not within other subsets, was significantly increased following MTT (Figure 6E). These results suggest that MTT induced the expansion of CX3CR1^+^GPR56^+^Ttr-like cells in HCC patients.

Next, we tracked the cluster distribution of expanded Ttr-like cells (increased >1.5-fold after MTT) before and after MTT based on TCR CDR3 nucleic acid sequences (Figure 6F). Our findings revealed that the majority of the TCR clonotypes (81.8%) that underwent clonal expansion maintained a consistent distribution of their subset before and after MTT (Figure 6G, upper). However, we found that four TCR clonotypes underwent subset shifts after MTT (Figure 6G, lower). These results suggest that, although these Ttr-like cells can undergo subpopulation switching, the increase in CX3CR1^+^GPR56^+^Ttr-like cells after MTT originates mainly from the expansion of resident subsets rather than from the conversion of other subsets.

### MTT-induced release of DAMPs activates cDCs to promote the expansion of Ttr-like cells

**Figure 7:**
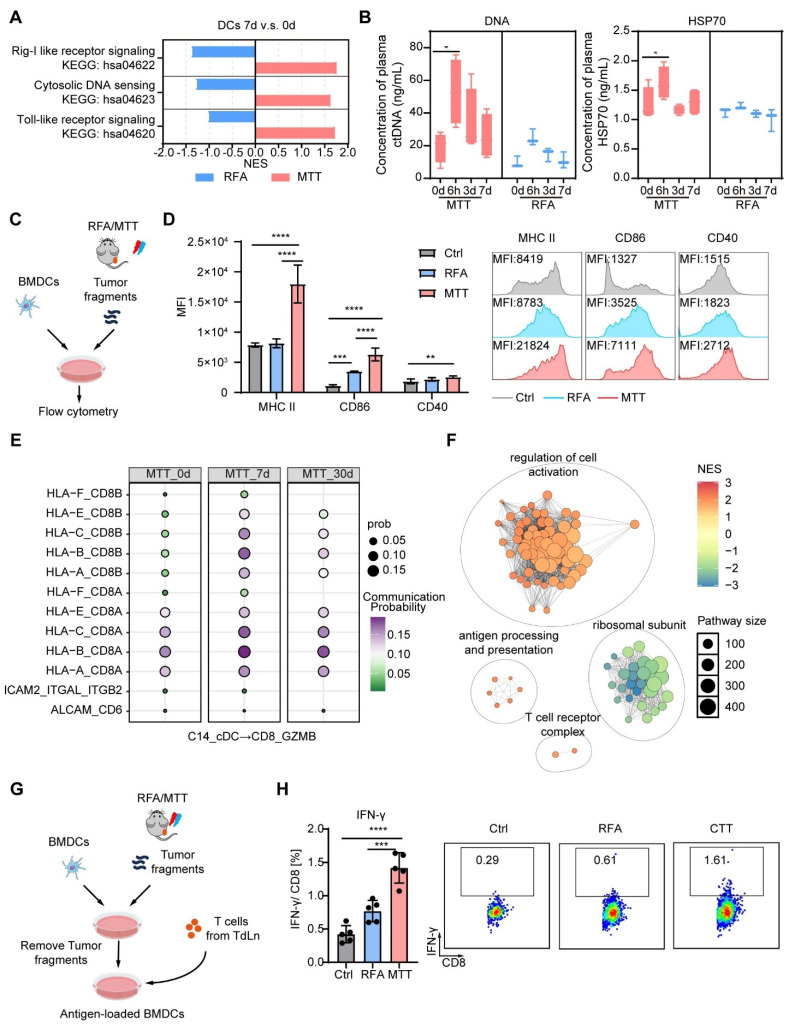
**MTT promotes the maturation of dendritic cells (DCs), thereby facilitating the expansion of Ttr-like cells.** (A) The NES of pathways associated to DAMPs sensing were identified using the R package fgsea along with the KEGG gene sets. (B) Serum concentrations of DNA and HSP70 in HCC patients were measured before and after MTT and RFA. n = 4 for MTT and n = 3 for RFA. One-way ANOVA was used. Error bars represent the standard deviation. *P < 0.05. (C) Schematic of the experimental design. Tumors harvested from mice after RFA or MTT treatment were minced and suspended in an equal mass of complete medium. Following centrifugation to remove large tissue debris and cells, the supernatant was co-cultured with BMDCs for 24 h. (D) Flow cytometry was used to detect the surface expression of MHCII, CD86, and CD40 on BMDCs. n = 5. (E) The probability of interaction of cDCs with clonally expanded Ttr-like cells was analyzed using the CellChat package. (F) Clustering network of significantly enriched Gene Ontology (GO) Biological Process (BP) terms identified through Gene Set Enrichment Analysis (GSEA) of clonally expanded Ttr-like cells 7 days after MTT. (G) Schematic of the ex vivo antigen presentation assay. Tumors harvested from mice after RFA or MTT treatment were minced and homogenized in an equal volume of complete medium. After centrifugation to remove large tissue debris and cells, the supernatant was co-cultured with BMDCs for 16 h to generate antigen-loaded BMDCs. The BMDCs were then harvested and co-cultured with T cells from tumor-bearing mice in the presence of brefeldin A (BFA) for 6 h. (H) Flow cytometry was used to detect intracellular IFN-γ expression in CD8⁺ T cells. n = 5. One-way ANOVA was used. Error bars represent the standard deviation. **P < 0.01, ***P < 0.001, ****P < 0.0001.

The generation of CX3CR1^+^ T cells depends on CD80/86 signaling from conventional dendritic cells (cDCs) and direct antigen interactions 17, 38. Our previous studies demonstrated that MTT causes extensive tumor cell necrosis, thereby promoting the release of DAMPs, including HSP70, HMGB1, calreticulin and nucleic acids 39, 40. DAMPs can be recognized by pattern recognition receptors and nucleic acid receptors on antigen-presenting cells, such as DCs, to promote their activation and antigen presentation 41. The KEGG gene set-based GSEA revealed that the toll-like receptor (TLR) signaling pathway (which may involve the binding of HSP70, HMGB1, and calreticulin), the cytosolic DNA sensing pathway, and the Rig-like receptor (a sensor for RNA) signaling pathway were significantly enriched in the cDCs of patients 7 days after MTT compared with those at baseline (Figure 7A).

However, these pathways were significantly downregulated in the cDCs of patients who received RFA (Figure 7A). Furthermore, MTT-induced cell death resulted in the rapid release of DAMPs, as evidenced by a significant increase in the plasma concentrations of DNA and HSP70 at 6 h posttreatment (Figure 7B). However, this increase was not observed in patients who received RFA (Figure 7B). Given these clinical findings, we next established a Hepa1-6 mouse model to investigate the direct effect of MTT- or RFA-released tumor-derived components on DC maturation. To this end, tumor fragments collected after MTT or RFA were co-cultured with bone marrow-derived DCs (BMDCs) for 24 h (Figure 7C). The results showed that components released post-MTT induced a significantly stronger upregulation of DC maturation markers (MHCII, CD86, and CD40) compared to those from RFA or control treatments (Figure 7D). In contrast, RFA-released components only induced a modest increase in CD86 expression relative to the control (Figure 7D).

To further investigate the mechanism by which MTT promoted the expansion of CX3CR1^+^ GPR56^+^ Ttr-like cells, receptor‒ligand interactions were explored between expanded CX3CR1^+^ GPR56^+^ Ttr-like cells and cDCs via CellChat. We observed significant upregulation of MHC-I-CD8A and MHC-I-CD8B interactions between cDCs and expanded Ttr-like cells at both 7 and 30 days post-MTT compared with baseline levels (Figure 7E). GSEA utilizing GO terms revealed significant enrichment of cell activation-related pathways in the expanded CX3CR1^+^ GPR56^+^ Ttr-like cell population compared with pretreatment levels (Figure 7F). Importantly, pathways involved in antigen presentation and T-cell receptor complex signaling were also markedly enriched (Figure 7F). To functionally validate these findings, we established an ex vivo antigen presentation assay. Briefly, tumor fragments from MTT- or RFA-treated mice were used to pulse BMDCs, which were subsequently co-cultured with T cells from tumor-bearing mice, enabling the assessment of DC-mediated T cell activation (Figure 7G). DCs pulsed with MTT-derived materials induced a significantly higher frequency of IFN-γ⁺ CD8⁺ T cells compared to DCs pulsed with RFA-derived materials or controls, confirming their superior antigen-presenting capability (Figure 7H). In summary, these findings suggest that tumor-derived components released after MTT effectively promote dendritic cell maturation and antigen presentation, thereby inducing the clonal expansion of tumor-reactive T cells.

The results presented earlier (as shown in Figure 2 E-F) indicated that the levels of Tregs were significantly decreased in PBMCs after MTT. Thus, we further performed CellChat analyses between cDCs and Tregs. We found that LGALS9-CD45 interactions between cDCs and Treg subpopulations were most significantly down-regulated (CD4_FOXP3 and CD4_LIMS1) (Figure S5). Galectin-9 encoded by LGALS9 can increase Treg stability and function through activation of Smad3 42. In addition, CD45 ligation specifically reduces Treg motility in an integrin-dependent manner, thereby enhancing Treg-DC interactions in vivo to promote Treg expansion and tolerance 43. Therefore, the reduced levels of Tregs in PBMCs would be attributed to the weakened LGALS9-CD45 interaction between cDCs and Tregs after MTT.

### MTT Elicits an Abscopal Effect in a Hepa1-6 Mouse HCC Model

**Figure 8:**
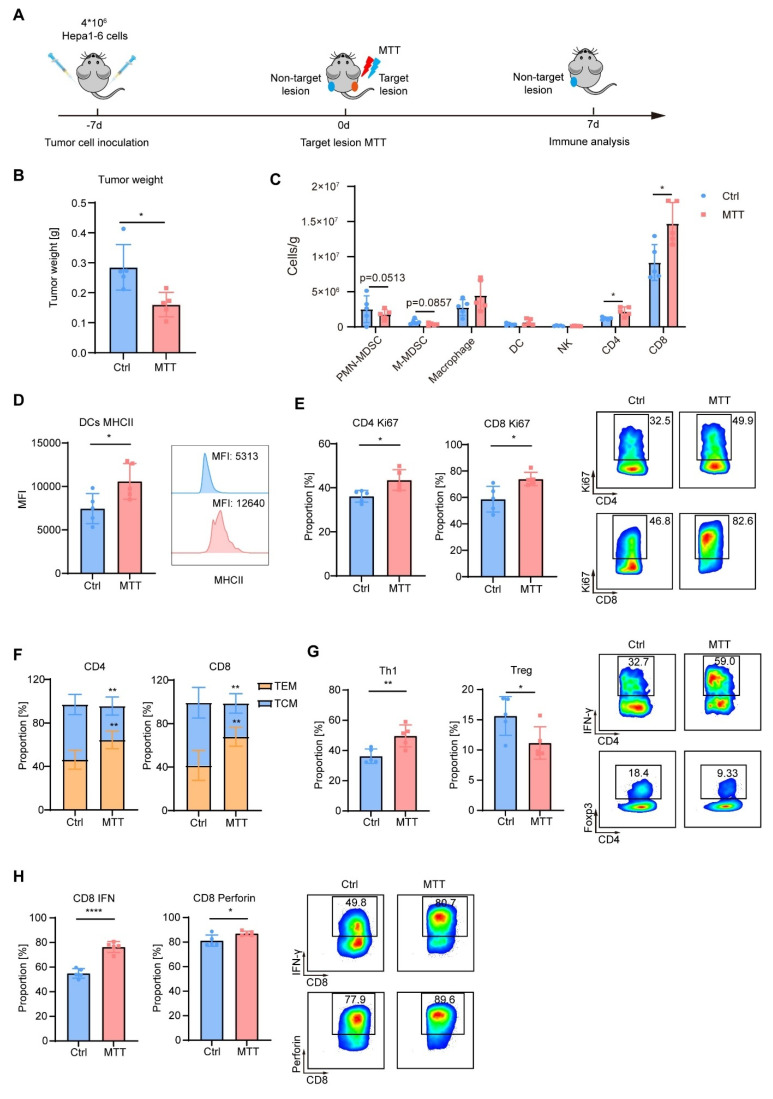
** MTT remodels the tumor immune microenvironment.** (A) Schematic of the experimental design. A bilateral Hepa1-6 tumor model was established, where the right tumor served as the target lesion receiving MTT, and the left tumor, as the non-target lesion, was analyzed by flow cytometry to assess changes in the tumor immune microenvironment. B) Tumor weight at the experimental endpoint. (C-H) Flow cytometric analysis of the non-target tumor: the numbers of PMN-MDSCs (CD11b⁺Ly6G⁺), M-MDSCs (CD11b⁺Ly6C⁺), macrophages (CD11b⁺F4/80⁺), DCs (CD11c⁺MHC II⁺), NK cells (CD3⁻CD49b⁺), CD4⁺ T cells (CD3⁺CD4⁺), and CD8⁺ T cells (CD3⁺CD8⁺) (C); MHC II expression on DCs (D); Ki67 expression in T cells (E); proportions of T_CM_ (CD62L⁺CD44⁺) and T_EM_ (CD62L⁻CD44⁺) cells (F); proportions of the Th1 subset (IFN-γ⁺) and Tregs (Foxp3⁺) (G); and expression of the effector molecules IFN-γ and perforin in CD8⁺ T cells (H). n = 5. Unpaired student's t-test was used. Error bars represent the standard deviation. *P < 0.05, **P < 0.01, ****P < 0.0001.

To further investigate the anti-tumor immunity induced by MTT, we established a bilateral Hepa1-6 tumor model in mice. In this model, the right-side tumor served as the target lesion receiving MTT, while the left-side tumor, as a non-target lesion, was monitored for changes in the tumor immune microenvironment 7 days after MTT (Figure 8A). We observed that compared to untreated control mice, the left-side tumors in MTT-treated mice showed a significant reduction in weight (Figure 8B). Compared with the control group, MTT decreased the numbers of PMN-MDSCs and M-MDSCs in the non-target tumors, while increasing the numbers of CD4^+^ T cells and CD8^+^ T cells (Figure 8C). Additionally, the expression of MHC class Ⅱ on DCs was significantly elevated in the MTT group compared to the control group (Figure 8D). Following MTT, Ki67 levels in both CD4^+^ T cells and CD8^+^ T cells were markedly increased (Figure 8E), indicating an active proliferative state of T cells. More importantly, the central memory subset (T_CM_, CD62L^+^CD44^+^) of both CD4^+^ and CD8^+^ T cells decreased, while the effector memory subset (T_EM_, CD62L^-^CD44^+^) significantly increased (Figure 8F). Compared to the control group, MTT led to an increase in the Th1 subset (IFN-γ^+^ cells) and a decrease in the Treg subset (Foxp3^+^ cells) among CD4^+^ T cells in the non-target tumor (Figure 8G). Furthermore, the expression of IFN-γ and perforin in CD8^+^ T cells was significantly enhanced (Figure 8H). These results demonstrate that MTT remodels the immune microenvironment of non-target tumors, thereby generating an abscopal effect.

## Discussion

Image-guided local thermal ablation, which is a noninvasive treatment option, plays an important role in the treatment of HCC. Local ablation therapies stimulate local and systemic antitumor immunity by inducing the immunogenic death of tumor cells. However, current ablation approaches induce weak and transient antitumor immunity leading to limited therapeutic efficacy. In this study, we evaluated the ability of MTT, a novel local ablative therapy, to trigger antitumor immunity in patients with HCC compared with conventional RFA. MTT but not RFA decreased Treg levels and increased the proportion of CX3CR1^+^GPR56^+^ effector T cells in the peripheral blood. These CX3CR1^+^GPR56^+^ effector T cells are characterized by high expression of cytotoxic molecules and are enriched with Ttr-like cells. Mechanically, MTT induced the release of DAMPs more than RFA, thereby activating cDCs and enhancing antigen presentation, which strengthened the interactions with CX3CR1^+^GPR56^+^ effector T cells via MHCⅠ-TCR, but weakened the interactions with Tregs via LGALS9-CD45, potentially explaining the observed clonal expansion of CX3CR1^+^GPR56^+^ Ttr-like cell populations, but the reduction of Tregs in the peripheral blood.

The combination of immune checkpoint blockade and bevacizumab, an anti-VEGF antibody, has received FDA approval as a first-line therapy for advanced HCC 44. However, the HCC TME exhibits strong immunosuppressive features 45, leading to limitations in drug response rates and the development of drug resistance. Indeed, this study revealed that HCC tumors exhibit strong immunosuppressive properties. We found that the TME of HCC patients was highly enriched with nonfunctional T cells and immunosuppressive Tregs. Furthermore, a considerable proportion of T cells with the potential to respond to tumors exhibit an exhausted, stress response phenotype. Therefore, remodeling the immunosuppressive environment of HCC tumors is particularly important.

A multitude of studies utilizing single-cell sequencing analysis have revealed that CX3CR1-expressing T cells have elevated cytotoxic and migratory scores and the highest degree of clonal expansion 31-33. These CX3CR1-expressing T_effs_ in the peripheral blood are a significant source of T_ex_ 31-33. In this study, we further validated the ability of CX3CR1^+^ T cells to produce cytotoxic molecules at the protein level; more importantly, we revealed their robust tumor reactivity in a Hepa1-6 mouse HCC model. The high level of expression of cytotoxic molecules and potent tumor reactivity of CX3CR1^+^GPR56^+^ T cells highlight their importance in antitumor immunity. Consistent with these findings, several studies have demonstrated that CX3CR1^+^ T cells exhibit enhanced tumor-killing capacity 46, 47. In the present study, MTT but not RFA increased the number of peripheral CX3CR1^+^ T cells in both HCC patients and animal models. Concurrently, the upregulation of CX3CR1^+^ T cells was prolonged in three of the four patients who underwent MTT, with the proportion maintaining a higher level than the basal level at 30 days following MTT. Owing to the increased cytotoxicity of CX3CR1^+^ T cells, CX3CR1^+^ T cells undergo activation-induced cell death, a process that can be prevented by anti-PD-1 antibodies 46. Therefore, the use of MTT in combination with immune checkpoint therapy may be a promising strategy for tumor treatment.

In a syngeneic mouse model of colon adenocarcinoma, neoantigen vaccination promoted the generation of circulating CX3CR1^+^CD8^+^ T cells, whose proportion exhibited a strong inverse correlation with tumor volume 38. Moreover, the expression of CX3CR1 on CD8^+^ T cells in the blood can be used as a marker of the response to immunotherapy, where a CX3CR1 score of ≥20 discriminates responders from nonresponders 17-19. We found that patients' CX3CR1 scores all increased after MTT, while three of the four patients' scores were much higher than 20 at both 7 and 30 days posttreatment (mean scores of 157.6 at 7 days and 101.3 at 30 days after MTT). CX3CR1 scores in PBMCs at 7 days after treatment were positively correlated with patient prognosis. These findings suggest that MTT as a local treatment may have immunostimulatory effects similar to those of immunotherapy. However, the sample size of this study was small. In future studies, more patient samples need to be collected for further validation.

The precise contribution of T cells to tumor-directed immunity is contingent upon the primary signals they receive via TCR engagement 16. Therefore, identifying markers for tumor-reactive T cells to assess the immune response during treatment is highly important. CXCL13 expression has been shown to effectively identify both exhausted and nonexhausted tumor reactive CD8^+^ T cell clones within the tumor 36. Nevertheless, our findings and those of other researchers indicate that T cells in PBMCs exhibit minimal expression of CXCL13 36. 4-1BB (CD137) is a significant marker of T cells that have encountered antigens 48. However, its expression is transient, which complicates its use as a marker for tumor-reactive T cells in PBMCs. Furthermore, studies have demonstrated that PD-1 and other inhibitory receptors exhibit limited efficacy as predictive markers of tumor-reactive T cells 49. Therefore, it is necessary to identify additional markers to distinguish tumor-reactive T cells in PBMCs. Our investigations revealed that MTT triggered the expansion of CX3CR 1^+^GPR56^+^ T cells within the PBMCs of patients. Notably, such cells have been previously documented as virus-specific T cells 50, 51. Consequently, TCR sequences associated with various pathologies and antigens were excluded prior to TCR analysis 34. We found that CX3CR1^+^GPR56^+^ T cells shared identical TCR sequences with expanded TCR clonotypes that were enriched in tumors. Additionally, by co-culturing tumor cells with autologous T cells, we demonstrated that the proportion of tumor-reactive T cells within CX3CR1⁺ T cells is significantly higher than that within CX3CR1⁻ T cells, both in the Hepa1-6 mouse liver cancer model and in HCC patients. Thus, we suggest that CX3CR1 and GPR56 could be markers for Ttr-like cells.

The efficacy of MTT stems from its optimized physical design to maximally induce immunogenic cell death, a process fundamental to adaptive anti-tumor immunity and less dependent on tumor histology. This is confirmed by its proven ability to enhance survival and drive potent T-cell responses across multiple preclinical tumor models (including colorectal, melanoma, breast, and lung cancers) and in patients with colorectal liver metastases 9-15, 52, 53. Notably, our unpublished data reveal that MTT consistently expands the peripheral CX3CR1⁺ T-cell pool in diverse mouse models (e.g., MC38, B16F10), mirroring our key finding in Hepa1-6 mice model and HCC patients. In summary, this collective evidence indicates that by inducing potent immunogenic cell death, MTT is capable of triggering a consistent and robust adaptive immune response across different tumor types. This is particularly manifested through the systemic expansion of the CX3CR1⁺ T-cell subset, thereby highlighting its significant potential as a broad-spectrum in situ tumor vaccination platform.

By promoting tumor cell fragmentation and the subsequent release of tumor antigens, MTT not only enhances the expansion of pre-existing Ttr cells but also drives the de novo generation of tumor neoantigen-specific T cells. However, owing to the challenges associated with studying newly generated tumor antigen-specific T cells, this study focused solely on analyzing the kinetics and subpopulation distributions of Ttr-like cells present in patients. In the future, the development of novel research tools will be essential to enable a more comprehensive evaluation of tumor-specific T cells following MTT.

The immunosuppressive environment of HCC results in a deficiency of tumor-reactive T cells with antitumor capabilities. Notably, our results revealed that many of the Ttr-like cells within the tumors were dysfunctional. This finding may explain why tumor-reactive T cells within tumors fail to inhibit tumor progression. In contrast, CX3CR1^+^GPR56^+^ T cells, which are Ttr cells, have high expression of cytotoxic molecules and low expression of coinhibitory molecules. At present, the majority of adoptive cell therapies involving tumor-infiltrating lymphocytes (TILs) employ T cells within the TME as the source of adoptive cell therapy. However, high enrichment of T_ex_ and Tregs may affect the therapeutic efficacy of TIL. In the future, the use of CX3CR1^+^GPR56^+^ tumor-reactive T cells derived from PBMCs as a cell source for ACT may increase the therapeutic efficacy of this treatment. Nevertheless, further research is necessary to optimize the enrichment strategy for tumor-responsive T cells in PBMCs.

In conclusion, our results suggest that MTT promotes cDC maturation by releasing more DAMPs and ultimately induces the expansion of CX3CR1^+^GPR56^+^ Ttr-like cells and reduces Treg levels compared with conventional RFA. This study elucidates the mechanism by which MTT induces systemic antitumor immunity and provides a theoretical basis for its combination with immunotherapies.

## Materials and Methods

### Patients and samples

This research protocol received ethical approval from the Ethics Committee of Fudan University Shanghai Cancer Center (No. 2108241-11), and all study procedures were conducted in compliance with the Declaration of Helsinki and Istanbul. Informed consent was obtained from all participants involved in this study. Seven patients with HCC were included, and their clinical information is summarized in Supplemental Table S1. The tumor biopsies were obtained before MTT using diagnostic needle biopsy with 18-G needles. The samples were washed with Hanks' balanced salt solution (HBSS) three times, minced into small pieces, and then digested with 3 mL of CelLive Tissue Dissociation Solution (Singleron) via the Singleron PythoN Tissue Dissociation System at 37 °C for 15 min. The cell suspension was collected and filtered through a 40-micron sterile strainer. Afterward, the GEXSCOPE® red blood cell lysis buffer (RCLB, Singleron) was used to remove red blood cells.

Blood samples were collected in EDTA tubes before MTT and on day 7 and 30 after MTT.

The PBMCs were isolated using Ficoll-Paque Plus medium (GE Healthcare), followed by washing with calcium- and magnesium-free PBS. Residual red blood cells were subsequently removed with 2 mL of GEXSCOPE® red blood cell lysis buffer (RCLB, Singleron). PBMCs were frozen in RPMI 1640 medium containing 10% human serum and 10% DMSO.

### Multimodal tumor thermal therapy and RFA procedure

Multimodal tumor thermal therapy (MTT) involves a sequential regimen that consists of rapid freezing, natural thawing, and radiofrequency (RF) heating applied to the targeted tumor tissue. This process achieves the precise combination of cryogenic and radiofrequency temperature fields, allowing for the in situ crushing of tumor cells and the subsequent release of immunoactive substances. In this clinical study, a comprehensive treatment modality was administered utilizing the multimodal tumor thermal therapy system (MTT-P1), developed by Magic-med in Shanghai, China. Guided by CT imaging, an integrated ablation needle (MTT-N1) with cryotherapy and RF heating was accurately inserted into the target tumor tissue. Subsequently, liquid nitrogen at a pressure of 1.0 MPa was precisely administered to the ablation needle via the MTT-P1 system to initiate the freezing process of the tumor. The freezing duration was controlled within 10 to 15 min until a sufficient iceball margin of no less than 5 mm relative to the tumor boundary was visually confirmed through CT imaging. Following the completion of the freezing process, a natural thawing phase lasting approximately 3 min took place to ensure complete melting of the iceball. RF heating was administered with a consistent temperature control strategy. The targeted temperature and heating duration were determined according to the manufacturer's recommended dose‒effect relationship and the dimensions of the tumor, thereby ensuring that a 50 °C temperature boundary enveloped the tumor with a safety margin. After the aforementioned MTT process was complete, the ablation needle was withdrawn while the needle tract ablation was simultaneously performed. A typical operation case, as shown in Figure S4, demonstrates the complete process of MTT. Additionally, image processing software (IMAGE-P1, Magic-med, Shanghai, China) was utilized to assist in precise temperature field control for the MTT. Its primary functions include cross-modal registration and fusion of preoperative MRI and intraoperative CT images, enabling more accurate tumor localization, needle positioning guidance, and ablation boundary evaluation, as shown in Figure S4C.

The Tumor Thermal-Immune Treatment System was employed in the treatment of animal models. The subcutaneous tumors of the mice were frozen with liquid nitrogen at -20 °C for 5 min and then heated with RF at 50 °C for 10 min.

RFA was performed using radiofrequency ablation electrode needles (MedSphere). All standard RFA procedures were carried out following the manufacturer's established guidelines.

### Cell lines and animal models

Murine Hepa1-6 cells were cultured in DMEM (MeilunBio) supplemented with 10% FBS (Gemini Bio-Products), 100 U/mL penicillin, and 100 μg/mL streptomycin (Hyclone).

Female C57BL/6 mice aged 6-8 weeks were sourced from Shanghai Slaccas Experimental Animal Co., Ltd (China). The animals were maintained in isolated cages under a 12 h light/dark cycle and provided with sterile nutritional feed and water. To establish the subcutaneous HCC mouse models, 6×10^6^ Hepa1-6 cells in 100 μL matrix gel were injected subcutaneously into the right femoral region of C57BL/6 mouse. For the establishment of the bilateral Hepa1-6 tumor model, 6×10^6^ Hepa1-6 cells in 100 μL matrix gel were injected subcutaneously into both the right and left femoral regions. All animal experiments were approved by the Animal Care and Use Committee of Shanghai Jiao Tong University and conducted in accordance with institutional guidelines.

### Single-cell RNA-sequencing, T cell repertoire profiling

Single-cell suspensions (2 × 10^5^ cells/ml) with PBS (HyClone, USA) were added to the microwell chip via a Singleron Matrix® single-cell processing system. The scRNA-seq library was subsequently established using the GEXSCOPE® Single-cell RNA Library Kit (Singleron), and the scTCR-seq libraries were constructed according to the protocol of the GEXSCOPE Single-cell Immuno-TCR/BCR Kit (Singleron Biotechnologies). Libraries for individuals were subsequently diluted to 4 ng/μL and pooled for sequencing by using Illumina HiSeq X (Illumina, San Diego, CA, USA) with 150-bp paired-end reads.

### Single cell data analysis and processing

The raw reads were processed to generate gene expression profiles using CeleScope v1.5.2 (Singleron Biotechnologies) with default parameters. Briefly, barcodes and UMIs were extracted from R1 reads and corrected. Adapter sequences and poly A tails were trimmed from R2 reads, and the trimmed R2 reads were aligned against the GRCh38 transcriptome using STAR (v2.6.1b). Uniquely mapped reads were then assigned to exons with FeatureCounts (v2.0.1). Successfully assigned reads with the same cell barcode, UMI and gene were grouped together to generate the gene expression matrix for further analysis.

The Seurat package (version 4.4.0) was used to analyze and process the gene expression matrix. Cells with gene counts between 300 and 4,000, UMI counts >1,000 and >35% mitochondrial genes were retained. Gene expression data were normalized and scaled using the NormalizeData and ScaleData functions, respectively. The top 2,000 variable genes were subjected to PCA, and the first 15 principal components were used for clustering. Cell clusters were finally visualized in two dimensions with UMAP.

The Seurat v4.0.0 FindMarkers function was utilized to identify differentially expressed genes (DEGs) of each cluster or each group with default parameters. DEGs were defined as genes that were expressed in more than 10% of the cells in a cluster and had no threshold for log fold change (logFC). The cell types were annotated based on the DEGs. DEGs were then ranked based on the average log fold change and used for gene set enrichment analysis (GSEA) using the R package fgsea (version 1.24.0). Pathways with adjusted p values < 0.05 were considered significantly enriched. The R package UCell (version 2.2.0) 54 was used to score each single cell by the gene signatures of naïve (*TCF7, CCR7, SELL* and* LEF1*), dysfunction (*PDCD1, CTLA4, TIGIT, HAVCR2, LAG3, LAYN* and* TOX)*, cytotoxic *(PRF1, IFNG, GZMA, GZMB, GZMH, GNLY, NKG7, KLRK1, KLRB1, KLRD1, CTSW* and* CST7*) 55 and Ttr (*CXCL13, CTLA4, ENTPD1, LAYN, TIGIT, BATF, HAVCR2, TNFRSF9* and* GZMB*) 36. The analyses of cell-cell communication were conducted with the R package CellChat 56 (version 1.6.1). The R package aPEAR 57 (version 1.0.0) was used for GSEA pathway network visualization.

### Calculation of CX3CR1 score

The CX3CR1 score is calculated based on the percentage increase in the proportions of CX3CR1⁺GPR56⁺CD4⁺ T cells and CX3CR1⁺GPR56⁺CD8⁺ T cells.

### Correlation between the CD8_GZMB/CD4_GZMB gene signature and patient prognosis in TCGA

The top 10 DEGs from CD4_GZMB and CD8_GZMB subsets were used to construct a gene signature. Liver hepatocellular carcinoma (LIHC) patients (n = 346) from TCGA were stratified into high- and low-expression groups based on median signature expression. Survival analysis was performed using Kaplan-Meier curves and log-rank tests.

### T cells migration in a transwell system

Hepa1-6 tumor cells were seeded in the bottom chamber for 12 h. Isolated CX3CR1^-^ T cells and CX3CR1^+^ T cells were placed in the upper compartment of a transwell chamber featuring uncoated polyester membrane with 5-µm pores (Sarstedt) for 5 h. The count of T cells in the bottom chamber was measured by flow cytometry using Precision Count Beads (BioLegend).

### Definition of Ttr-like cells

TCR clonotype assignment was performed using the Cell Ranger (v4.0.0) vdj pipeline with GRCh38 as a reference. In brief, a TCR diversity metric, containing clonotype frequency and barcode information, was obtained. Each unique TCR β-chain was defined as a clonotype. TCR clonotypes were defined as TCRs with the same TRB-CDR3 nucleotide sequences. TCR clonotypes that commonly recognize bacterial, viral, and autoantigens were removed on the basis of the TRB-CDR3 sequences 34. TCR clonotypes with numbers > 5 in the tumor microenvironment (TME) were subsequently defined as Ttr-like cells. The R package immunarch (version 0.9.1) was used to analyze the TCR repertoire overlap. Immunomap 35 is used to analyze the structural similarity of the TCR repertoire, the Shannon-Weiner index, and the response contributed by single clones.

### Flow cytometry

Tumor tissues from mice were minced and digested into single-cell suspensions using a cocktail of collagenase I (Yeasen), hyaluronidase (Yeasen), and DNase I (Sigma). Red blood cells were subsequently removed using a red blood cell lysis buffer. Information on the antibodies used for flow cytometry is presented in Table S3. Surface antibody staining was performed at 4 °C for 30 min. Cells were treated with either a Cell Activation Cocktail (BioLegend) or brefeldin A (BioLegend) following the manufacturer's protocol prior to intracellular staining. Live/dead discrimination was carried out with the Zombie Aqua™ Fixable Viability Kit (BioLegend). After surface staining with antibodies, cells were fixed and permeabilized using Fixation Buffer (BioLegend) and Intracellular Staining Permeabilization Wash Buffer (BioLegend). Intracellular cytokines were then stained with corresponding antibodies at 4 °C for 30 min. Staining for Ki67 and FoxP3 was performed using the True-Nuclear™ Transcription Factor Buffer Set (BioLegend) according to the manufacturer's instructions. Cell fluorescence was assessed with an LSRFortessa (BD Biosciences, USA) and analyzed with FlowJo software (version 10.6.2).

### Tumor-reactive T cells assay

To detect tumor-reactive T cells in mice hepa1-6 model, splenocytes from mice treated with MTT were cocultured with Hepa1-6 cells for 12 h and then treated with brefeldin A for 6 h. Intracellular IFN-γ expression was then detected by flow cytometry.

To assess tumor-reactive T cells in the PBMCs of HCC patients, tumor tissues were collected and digested with collagenase I (Yeasen), hyaluronidase (Yeasen), and DNase (Sigma) to generate single-cell suspensions. Intratumoral CD45^+^ leukocytes were subsequently depleted using an APC Positive Selection Kit II (STEMCELL). The tumor cell suspension was co-cultured with autologous PBMCs for 12 h followed by a 6 h incubation with brefeldin A. Intracellular IFN-γ expression in T cells was finally analyzed by flow cytometry.

### Measurement of plasma DNA and HSP70 concentrations

The concentration of DNA in the plasma was measured by a dsDNA HS Assay Kit (Yeasen) according to the manufacturer's instructions. The concentration of DNA in the plasma was measured with an HSP70 High Sensitivity ELISA Kit (Abcam).

### Preparation, maturation, and functional characterization of BMDCs

Bone marrow cells were harvested from the femurs of C57BL/6 mice. The cells were cultured in complete RPMI-1640 medium containing 20 ng/mL recombinant murine GM-CSF (Novoprotein) and 10 ng/mL recombinant murine IL-4 (Novoprotein).

To evaluate the effect of tumor-derived components released post-ablation on dendritic cell maturation, tumors were harvested from mice after MTT or RFA treatment. The tumor tissue was minced and homogenized in an equal volume of complete medium. Following thorough vortexing, the homogenate was centrifuged at 400 × g for 10 min. The supernatant was collected, and this centrifugation step was repeated twice to remove cellular debris. The resulting supernatant was then co-cultured with BMDCs for 24 h. Subsequently, the expression of DC maturation markers (MHCII, CD86, and CD40) was analyzed by flow cytometry.

To investigate the stimulatory effect of antigen-loaded BMDCs on tumor-reactive T cells, tumor antigen-containing supernatant was prepared according to the aforementioned procedure and used to pulse BMDCs for 16 h. After removing the supernatant by washing, these BMDCs were co-cultured with T cells from tumor-bearing mice in the presence of brefeldin A (BFA) for 6 h. IFN-γ expression in CD8⁺ T cells was subsequently assessed by flow cytometry.

### Statistical analysis

The results are expressed as the mean ± standard deviation (SD). Statistical analyses were conducted with Student's t-test (for unpaired data), paired Student's t-test (for matched observations) or one-way ANOVA (for comparisons among more than two groups). A P value of < 0.05 was regarded as statistically significant.

## Supplementary Material

Supplementary figures and tables.

## Abbreviations

MTT: Multimodal tumor thermal therapy
HCC: hepatocellular carcinoma
RFA: radiofrequency ablation
MWA: microwave ablation
DAMPs: damage-associated molecular patterns
cDC1s: conventional type I dendritic cells
CRCLM: colorectal liver metastases
PBMC: peripheral blood mononuclear cell
TACE: transcatheter arterial chemoembolization
PFSs: progression-free survival
TLR: toll-like receptor
GSEA: Gene set enrichment analysis
TME: tumor microenvironment
scTCR-seq: single-cell T-cell receptor sequencing
TILs: tumor-infiltrating lymphocytes
