# Supplementary material for: Multimodal tumor thermal therapy enhances antitumor immunity by expanding tumor-reactive CX3CR1⁺GPR56⁺ T cells in hepatocellular carcinoma
Source: Theranostics. 2026 Feb 26;16(9):4959–79. doi: 10.7150/thno.127962 (PMC12964383; doi:10.7150/thno.127962)
Supplement: Supplementary file 1 — Supplementary figures and tables. [file thnov16p4959s1.zip › Supplemental material/Figure S1-S6.docx]

**Supplemental material**


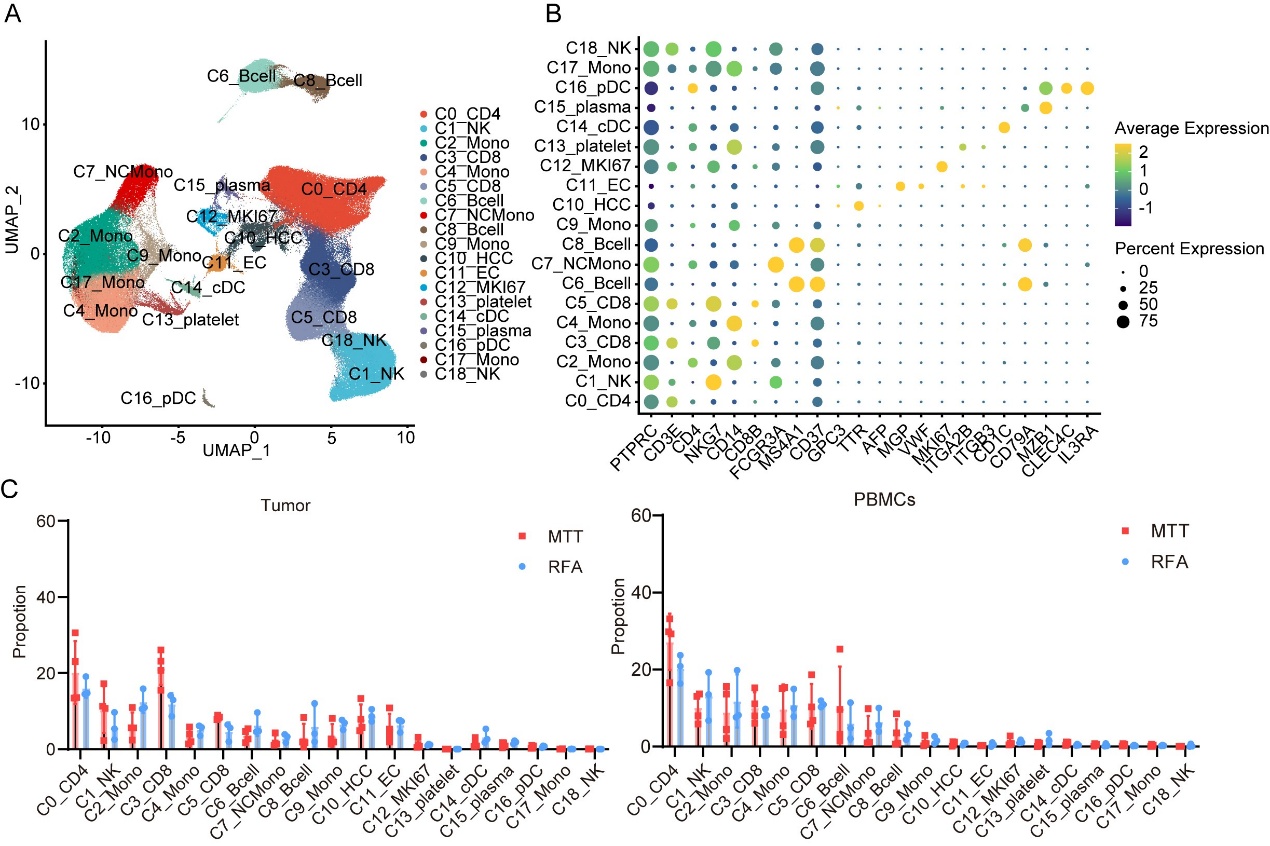


**Figure S1.** **Immune cell landscape of peripheral blood mononuclear cells (PBMCs) and tumors in HCC patients.** (A) UMAP visualization of cells transcriptomes in tumor and PBMCs from HCC patients. (B) The expression levels of the signature gene for each cluster. (C) Proportions of various immune cell subsets in the preoperative tumor microenvironment and peripheral blood of patients before MTT or RFA.


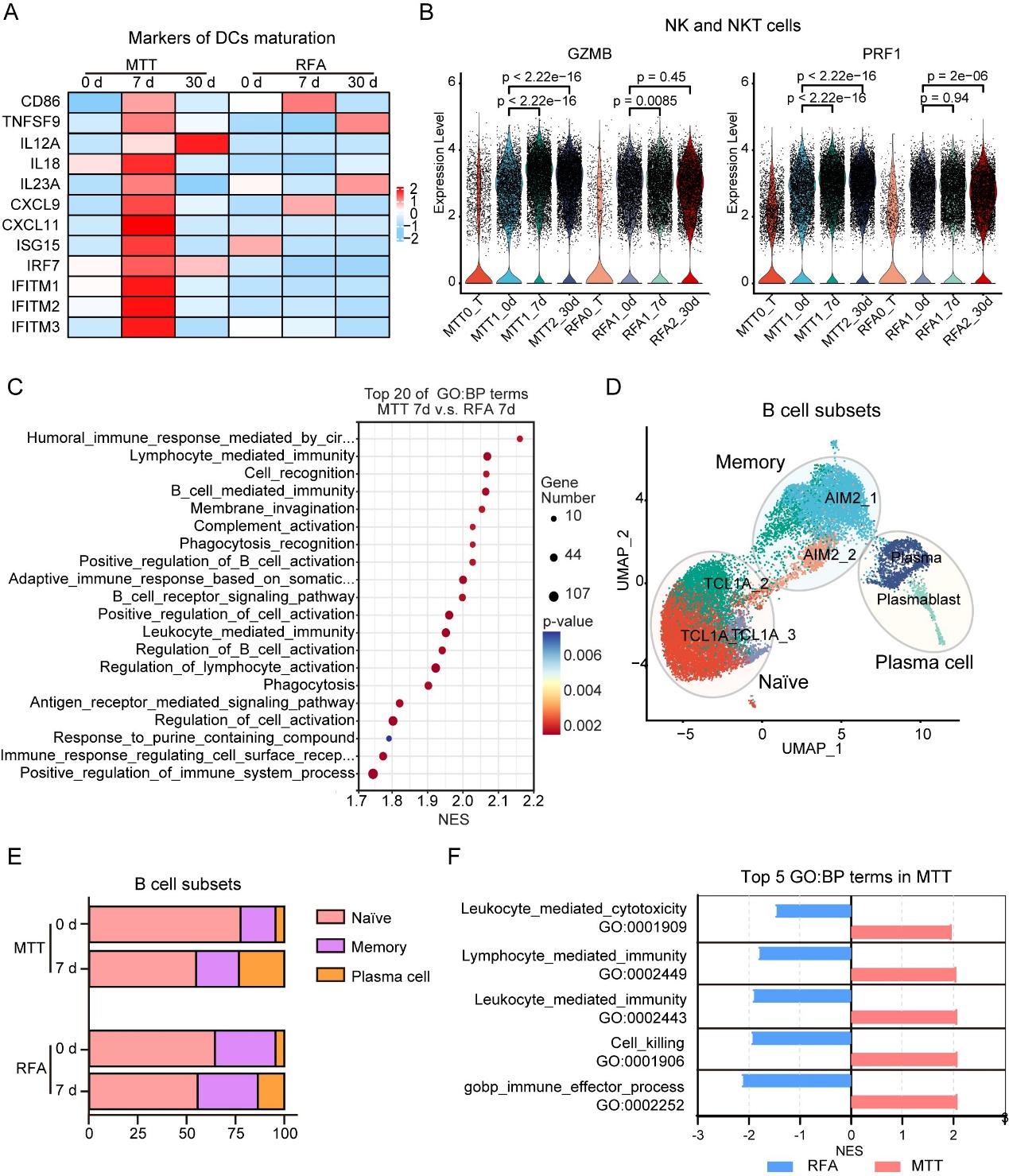


**Figure S2. MTT promoted the maturation of DCs, NK cell cytotoxicity, and plasma cell differentiation.** (A) Heatmap showing the expression levels markers associated with the maturation of DCs. (B) Violin plots showed the expression levels of *GMZB* and *PRF1* in NK cells from tumor and in PBMCs at different time points. The P-values were calculated using R package of ggsignif (version 0.6.4). (C) The top 20 upregulated pathways of B cells 7 days after the MTT versus B cells at baseline in PMBCs were identified using the R package fgsea along with the GOBP gene sets. (D) UMAP visualization of B cells transcriptomes in tumor and PBMCs from HCC patients. (E) The proportion of naïve B cells (B_TCL1A_1, B_TCL1A_2 and B_TCL1A_3), memory B cells (B_AIM2) and plasma cells (Plasma cell and Plasmablast) from PBMCs at baseline, 7 days and 30 days after MTT or RFA. Student’s two tailed t-test was used. *P < 0.05. (F) The top 5 upregulated pathways of T cells 7 days in MTT group versus T cells at RFA group in PMBCs were identified using the R package fgsea along with the GOBP gene sets.


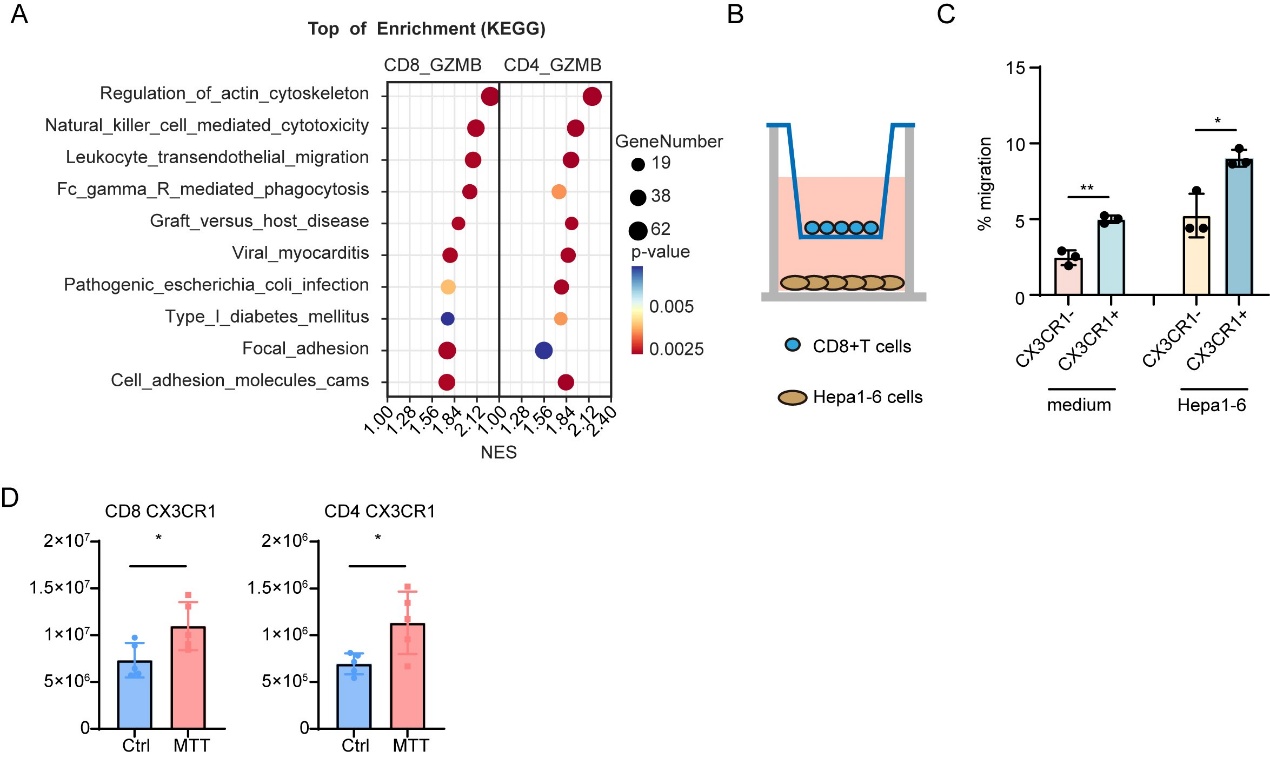


**Figure S3. MTT promoted the infiltration of CX3CR1⁺ T cells into tumors.** (A) The top 10 upregulated pathways in clusters of CD8_GZMB and CD4_GZMB versus other clusters were identified using the R package fgsea along with the KEGG gene sets. (B) Schematic diagram of the co-culture. CX3CR1^-^CD8^+^ T cells and CX3CR1^+^CD8^+^ T cells were labeled with CFSE and co-cultured with or without Hepa1-6 tumor cells in a transwell system for 5 h. The number of T cells in the lower chamber was measured using flow cytometry. (C) The migration ratio was calculated by the ratio of the number of T cells in the lower chamber to the original number of T cells in the upper chamber. n=3. Unpaired student’s t-test was used. (D) A bilateral Hepa1-6 tumor model was established, where the right tumor served as the target lesion receiving MTT, and the left tumor, as the non-target lesion, was analyzed by flow cytometry to assess the numbers of CX3CR1^+^T cells 7 days after MTT. n=5. Unpaired student’s t-test was used. *P < 0.05, **P < 0.01.


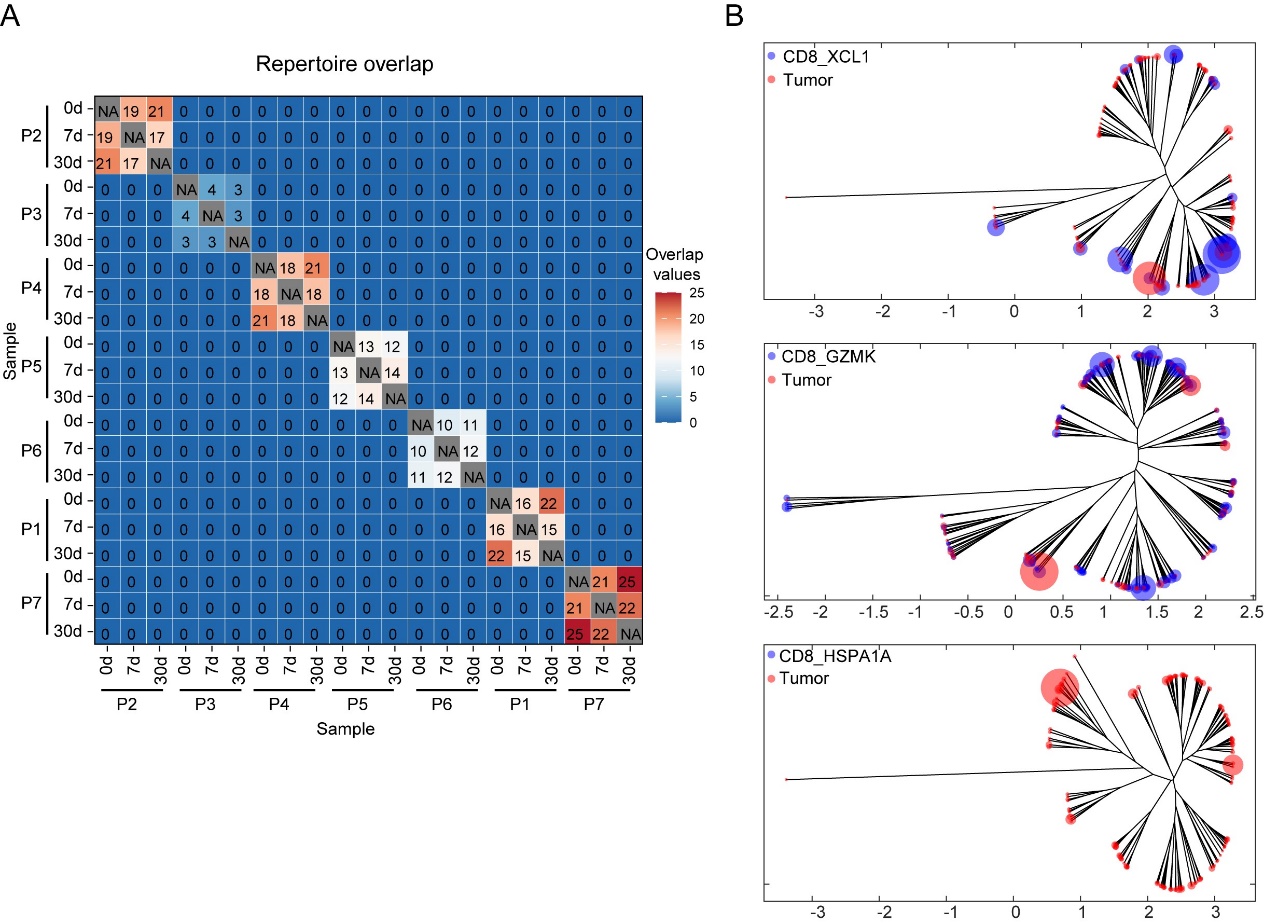


**Figure S4. The TCR repertoire of tumor tissues and PBMCs.** (A) No shared T-cell receptor (TCR) clonotypes were observed across patients. (B) Overlapping weighted repertoire dendrograms of TCR repertoires between PBMC clusters and tumor. (Red = Tumor repertoire; Blue = PBMC cluster repertoires).


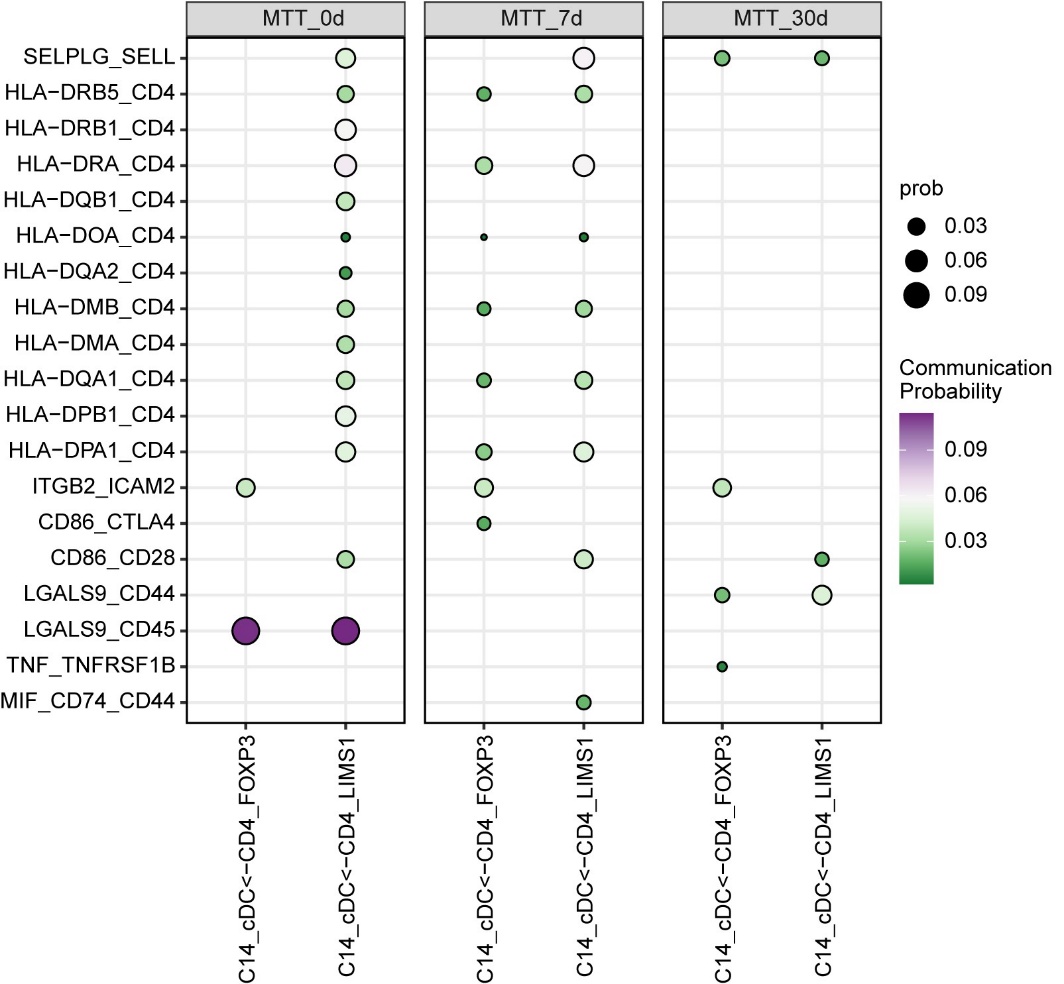


**Figure S5. LGALS9-CD45 interactions between cDCs and Treg subpopulations were significantly downregulated after MTT.** The probability of interaction of cDCs with Tregs was analyzed using the CellChat package.


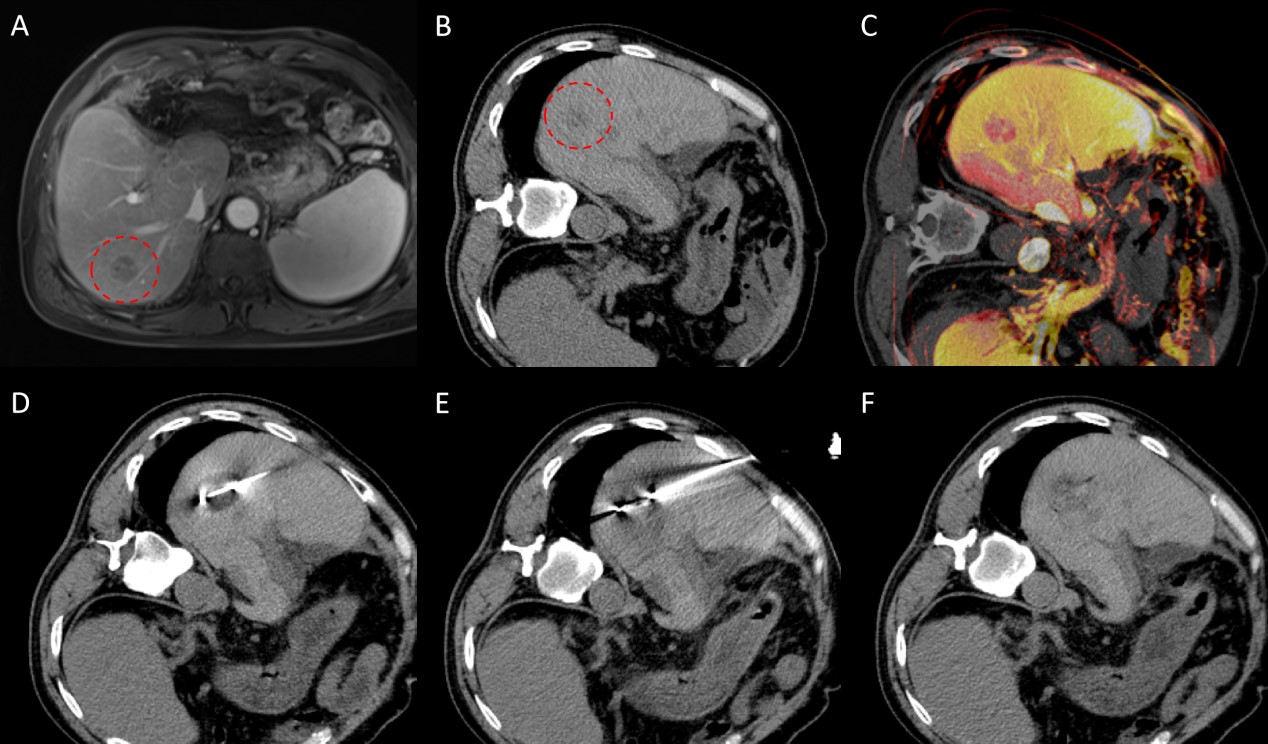


**Figure S6. Operational procedure of the Multimodal Tumor Thermal Therapy: a typical case.** (A) The preoperative MRI; (B) The intraoperative CT images; (C) Cross-modal registration and fusion of preoperative MRI and intraoperative CT images; (D) CT images after 15 minutes of freezing process; (E) CT images after 15 minutes of RF heating at 95℃; (F) Post-ablation CT images.
