# Supplementary material for: Multimodal tumor thermal therapy enhances antitumor immunity by expanding tumor-reactive CX3CR1⁺GPR56⁺ T cells in hepatocellular carcinoma
Source: Theranostics. 2026 Feb 26;16(9):4959–79. doi: 10.7150/thno.127962 (PMC12964383; doi:10.7150/thno.127962)
Supplement: Supplementary file 1 — Supplementary figures and tables. [file thnov16p4959s1.zip › Supplemental material/Table S1.docx]

| **Table S1. Clinical information of five included HCC patients** | | | | |
| --- | --- | --- | --- | --- |
| Patient ID | Group | History of prior treatment | Tumor size(cm) | PFS (days) |
| P1 | MTT | Surgery | 2.2*2.2 | 180 |
| P2 | MTT | Surgery, TKI | 1.38*1.41 | 540 |
| P3 | MTT | Surgery | 1.5*1.5 | 360 |
| P4 | MTT | Surgery, TACE, TKI | 2.15*2.41 | 382 |
| P5 | RFA | TACE, Cryo-ablation, ICIs, | 2.41*2.61 | 90 |
| P6 | RFA | Surgery | 2.07*1.72 | 30 |
| P7 | RFA | TACE 2× | 2.2*1.7 | 180 |
